# DNA Damage in Mammalian Neural Stem Cells Leads to Astrocytic Differentiation Mediated by BMP2 Signaling through JAK-STAT

**DOI:** 10.1016/j.stemcr.2013.06.004

**Published:** 2013-07-25

**Authors:** Leonid Schneider, Serena Pellegatta, Rebecca Favaro, Federica Pisati, Paola Roncaglia, Giuseppe Testa, Silvia K. Nicolis, Gaetano Finocchiaro, Fabrizio d’Adda di Fagagna

**Affiliations:** 1 IFOM Foundation–The FIRC Institute of Molecular Oncology Foundation, Via Adamello 16, 20139 Milan, Italy; 2 Fondazione I.R.C.C.S Istituto Neurologico C. Besta, Via Celoria 11, 20133 Milan, Italy; 3 European Institute of Oncology, Via Adamello 16, 20139 Milan, Italy; 4 Department of Biotechnology and Biosciences, University of Milano-Bicocca, Piazza della Scienza 2, 20126 Milan, Italy; 5 Neurobiology Sector, SISSA, Via Bonomea 265, 34136 Trieste, Italy; 6 Istituto di Genetica Molecolare CNR, Via Abbiategrasso 207, 27100 Pavia, Italy

## Abstract

The consequences of DNA damage generation in mammalian somatic stem cells, including neural stem cells (NSCs), are poorly understood despite their potential relevance for tissue homeostasis. Here, we show that, following ionizing radiation-induced DNA damage, NSCs enter irreversible proliferative arrest with features of cellular senescence. This is characterized by increased cytokine secretion, loss of stem cell markers, and astrocytic differentiation. We demonstrate that BMP2 is necessary to induce expression of the astrocyte marker GFAP in irradiated NSCs via a noncanonical signaling pathway engaging JAK-STAT. This is promoted by ATM and antagonized by p53. Using a SOX2-Cre reporter mouse model for cell-lineage tracing, we demonstrate irradiation-induced NSC differentiation in vivo. Furthermore, glioblastoma assays reveal that irradiation therapy affects the tumorigenic potential of cancer stem cells by ablating self-renewal and inducing astroglial differentiation.

## Highlights

• Irradiated NSCs undergo niche-independent cellular senescence and differentiation
• Astrocytic differentiation depends on cytokine secretion controlled by ATM and p53
• Induction of astrocytic marker GFAP requires noncanonical BMP2/JAK-STAT signaling
• Irradiation-induced differentiation occurs in vivo in SVZ-NSC and murine GBM model

## Introduction

The relationship between cell-cycle control and regulation of differentiation is a major question in stem cell biology. Neural stem cells (NSCs) are among the best characterized mammalian stem cells; they generate the central nervous system during development and support adult neurogenesis throughout life in the subventricular zone (SVZ) and subgranular layer of the hippocampus (Bonfanti and Peretto, 2007; Doetsch, 2003).

NSCs were the first somatic stem cell type shown to grow indefinitely in vitro under self-renewing conditions as neurospheres (Reynolds and Weiss, 1992). NSC cultures can be derived ex vivo from both the developing and adult brain or from embryonic stem (ES) cells and can differentiate into the three brain lineages: neurons, astrocytes, and oligodendrocytes (Conti et al., 2005; Pollard et al., 2006). This differentiation is governed by extracellular ligands and cytokines (Gangemi et al., 2004) and is associated with the downregulation of NSC markers such as Nestin, SOX2, and PAX6 (Conti et al., 2005; Gómez-López et al., 2011). Self-renewing cells with gene expression patterns similar to normal NSCs can also be found in glioblastoma multiforme (GBM), supporting the concept of cancer stem cells (Nicolis, 2007).

We recently showed that the canonical DNA damage response (DDR) signaling pathways (Figure S1A available online) are functional in NSCs (Schneider et al., 2012). Generation of DNA double-strand breaks (DSBs), e.g., by ionizing radiation, leads to activation and focal recruitment of the apical PI3K-like serine/threonine kinase (ATM), which labels chromatin at DNA lesions through phosphorylation of the histone H2A variant H2AX (γH2AX). ATM also phosphorylates the serine/threonine-glutamine (S/TQ) motif of many downstream effectors, some of which are focally recruited at DSBs (e.g., 53BP1), whereas kinases and transcription factors like CHK2 and p53 further relay DDR signaling, causing transient cell-cycle arrest to allow DNA repair or, depending on the nature of the DNA damage, apoptosis or cellular senescence (d’Adda di Fagagna, 2008; Jackson and Bartek, 2009; Shiloh, 2006).

## Results

### DNA Damage in NSCs Leads to Cellular Senescence Despite Transcriptional Downregulation of DDR Signaling

**Figure 1:**
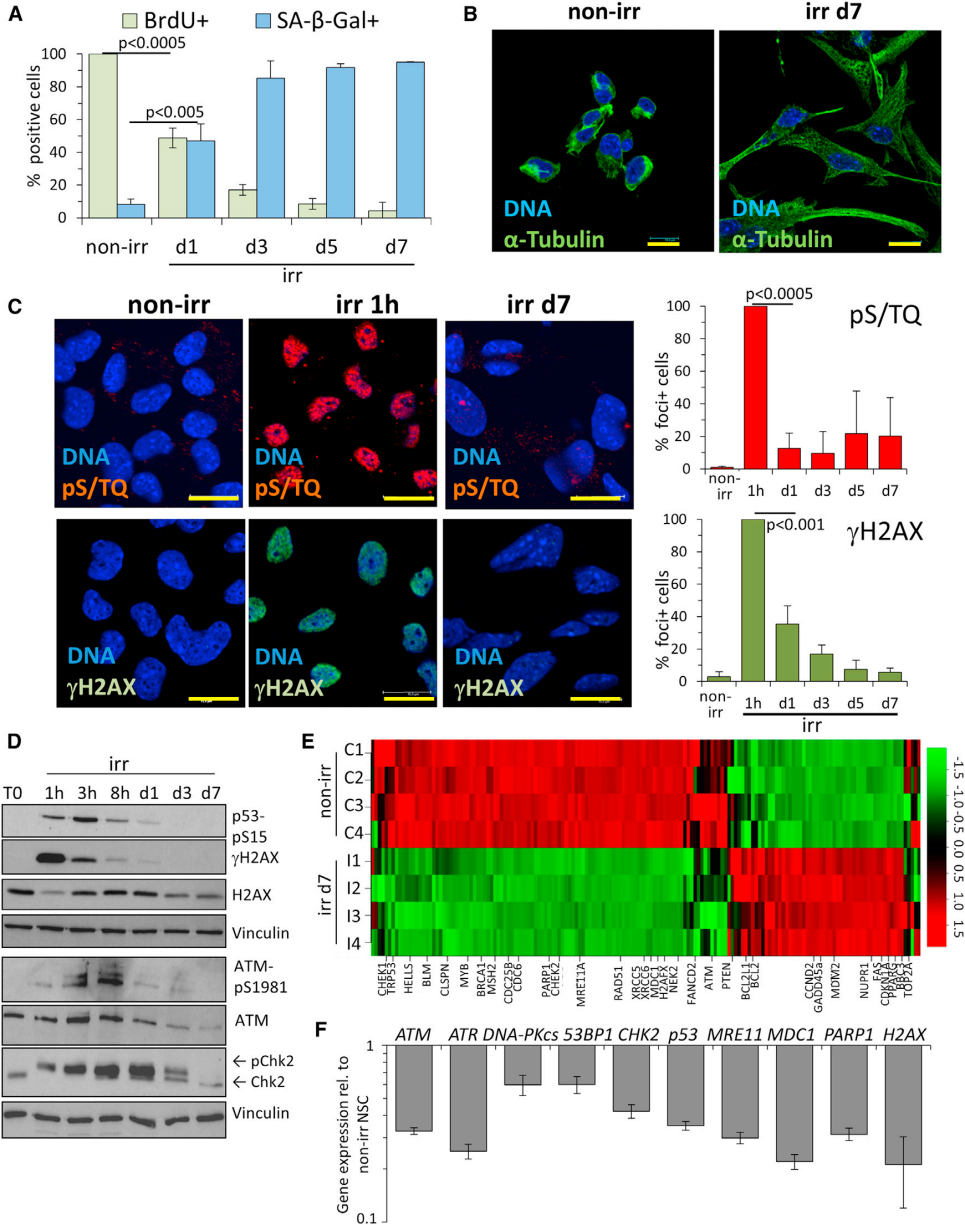
X-Ray Irradiation of NSC Leads to a Senescence-like Cell-Cycle Arrest, Associated with Progressive Downregulation of DDR Signaling (A) Time-course study of DNA replication and cellular senescence in nonirradiated (non-irr) and irradiated NSCs (irr) by wide-field microscopy. BrdU was supplied 24 hr prior to fixation and BrdU-incorporating cells were detected by immunofluorescence (IF). Senescent NSCs were analyzed by senescence-associated β-galactosidase (SA-β-Gal) activity assay and scored as positive for the characteristic blue signal. Error bars show SD. (B) Representative confocal images showing changes in cell morphology in irr NSCs; cytoskeleton was visualized by α-tubulin IF. Bar: 15 μm. (C) One hour after irr, NSCs uniformly display ATM/ATR kinase activity as demonstrated by the detection of foci of the typical phospho-epitope (pS/TQ, upper panel, red) and the phosphorylated histone H2AX (γH2AX, lower panel, green), as analyzed by confocal microscopy of IF stainings. Bar: 15 μm. Quantifications of DDR-positive cells are provided on the right-hand side. (D) Western blot (WB) analysis of DDR signaling in NSCs after irr. DDR activation was detected by autophosporylated ATM (phospho-serine 1981), mobility shift of CHK2, p53 phosphorylation at ATM-dependent site (serine 15), and γH2AX. (E) Microarray analysis and heatmap of DDR and cell-cycle control genes from gene sets obtained from Gene Ontology classes and literature (Jackson and Bartek, 2009). Relevant genes are highlighted. (F) Quantitative real-time PCR analysis of NSCs on day 7 post-irr for the expression of key DDR and DNA repair genes. Error bars show SD. See also Figure S1.

We induced DSBs in proliferating self-renewing NSCs, which uniformly display all key features of radial glia (Conti et al., 2005), by acute exposure to 10 Gy X-ray irradiation (irr). Many cells survived and exited the cell cycle, as indicated by reduced bromodeoxyuridine (BrdU) incorporation (Figure 1A) and expression of cell-cycle arrest markers such as p21^CIP^, p27^KIP^, and Rb-dephosphorylation (Figure S1B). Within 24 hr of irr, most and, after 3 days, all NSCs became enlarged with flattened morphology and expressed senescence-associated β-galactosidase (SA-β-gal) activity (Figures 1B and S1C). Upon DNA damage, such dramatic changes are usually associated with cellular senescence, commonly requiring continuous DDR signaling (d’Adda di Fagagna, 2008). Unexpectedly, whereas DDR signaling was promptly activated in NSCs immediately after irr (Figures 1C and S1D), it was progressively lost in the majority of cells entering senescence, as determined by DDR foci detection at the single-cell level for the DDR markers pS/TQ, γH2AX (Figure 1C), phospho-ATM, and 53BP1 (Figure S1D). Progressive reduction in DDR signaling was confirmed by immunoblotting for γH2AX, phospho-ATM, phospho-Chk2, and phospho-p53 (Figure 1D). Reduction in DDR foci is usually interpreted as accomplished DNA repair, including in NSCs (Acharya et al., 2010), and indeed we confirmed DSB repair proficiency in irr NSCs (Figure S1E). Yet, we noticed that the progressive loss in detectable phospho-ATM and its target phospho-CHK2 correlated with reduced expression of total ATM and CHK2 proteins in irr cells (Figure 1D). We then performed microarray analyses on control NSCs and NSCs 7 days after irr. In irr NSCs, we detected gene expression changes associated with cell-cycle arrest (*p21*^*CIP*^, *PTEN*, *GADD45α*, *CDC6*, *CDC25B*) but also a widespread downregulation of DDR genes (Figure 1E). The transcriptional downregulation of selected key DDR genes (*ATM*; *CHK2*, *p53*, *MDC1*, *H2AX*) was confirmed by quantitative real-time PCR (Figure 1F). Our study thus revealed a form of DNA-damage-induced cellular senescence maintained in the absence of a functional DDR signaling.

### DNA Damage Induces Astrocytic Differentiation

**Figure 2:**
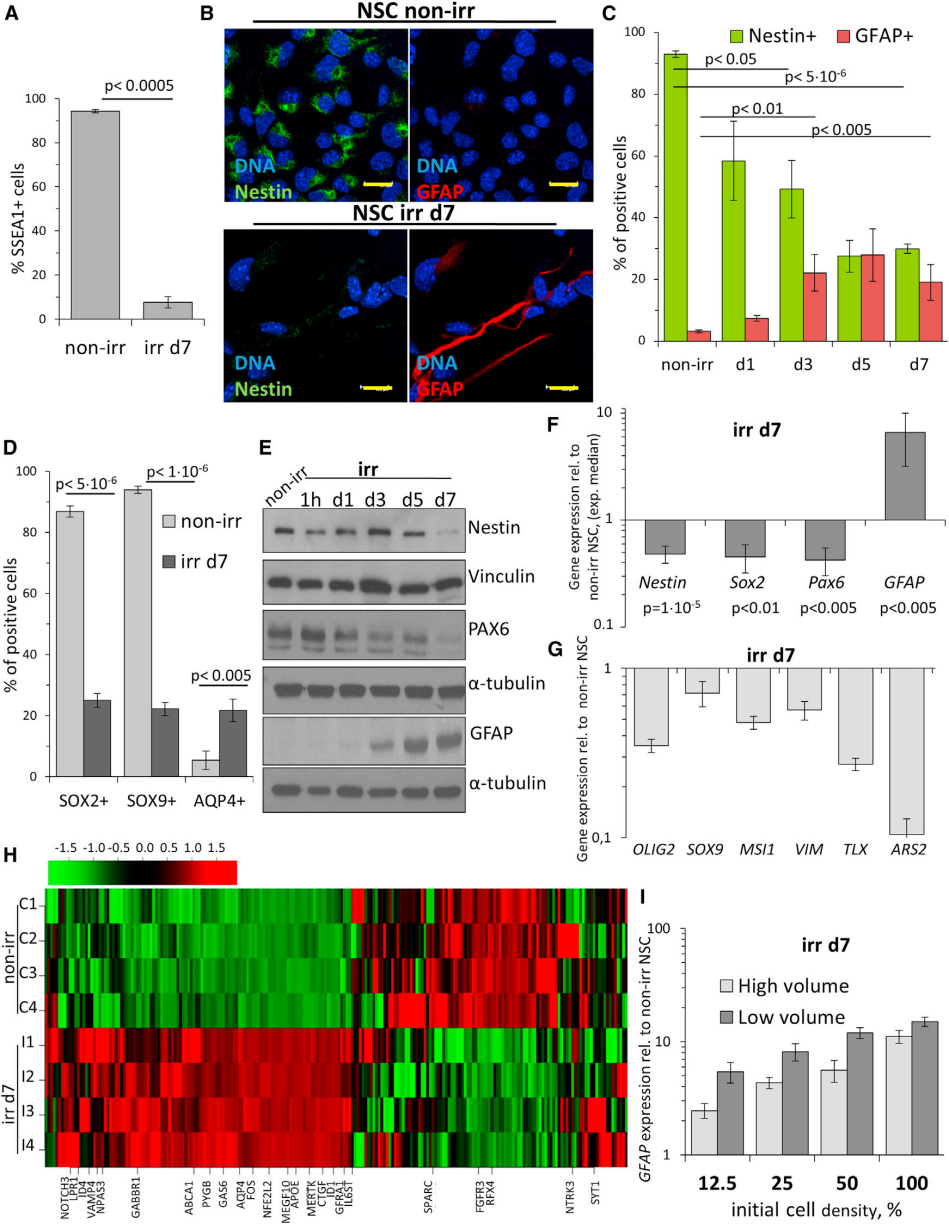
Irradiation Leads to Downregulation of Stem Cell Markers and Differentiation toward the Astrocytic Lineage (A) Quantification of the expression of NSC-typical membrane marker SSEA-1 in non-irr and irr cells at day 7 as detected by immunostaining and flow cytometry. Error bars show SD. (B) Representative confocal images showing the NSC-typical filament Nestin and the astrocyte filament GFAP prior and 7 days after irr, as detected by double IF analysis. Bar: 15 μm (C) Quantification of a time-course study of Nestin and GFAP expression in irr NSCs as detected by IF and wide-field microscopy. Error bars show SEM. (D) Quantification of the nuclear signal of NSC transcription factors SOX2 and SOX9 as well as the expression of astrocyte-typical membrane channel Aquaporin4 (AQP4) in non-irr and irr NSCs as detected by IF and wide-field microscopy. Error bars show SD. (E) WB analysis of stem cell (Nestin, PAX6) and astrocyte (GFAP) relevant protein expression upon irr of NSCs. (F) Median values of six or more experiments, analyzed by quantitative real-time PCR for the expression of NSC markers *Nestin*, *SOX2*, and *PAX6* and the astrocyte marker *GFAP*. Error bars show SEM. (G) Quantitative real-time PCR analysis of NSCs at day 7 post-irr showing downregulation of typical NSC markers: *OLIG2*, *SOX9*, *Musashi1* (*MSI1*), *Vimentin* (*VIM*), *TLX*, and *ARS2*. Error bars show SD. (H) Microarray analysis and heat map of genes reported as upregulated in brain-derived astrocytes and astrocytes produced from NSCs through serum exposure (Cahoy et al., 2008; Obayashi et al., 2009). Relevant astrocyte genes as mentioned in Cahoy et al. (2008) are highlighted. (I) NSCs were irradiated at different cell densities and medium replaced with 1× volume (low) or 2× volume (high). The expression of *GFAP* was analyzed by quantitative real-time PCR. Error bars show SD. See also Figure S2.

According to our previous report (Schneider et al., 2012), these findings may indicate that irr NSCs had lost their stem cell features and committed to an astrocytic fate. When we investigated the expression of the self-renewal-associated cell membrane marker SSEA1/LeX (Capela and Temple, 2006), only a small fraction of irr NSCs at day 7 stained positive, whereas all control cells did (Figure 2A).

We also detected a progressive loss of NSC-associated cytoskeletal filament Nestin in individual senescent NSCs and the appearance of cells expressing the astrocyte-associated intermediate filament GFAP (Figures 2B, 2C, and S2A). Next, we quantified the nuclear signal of the key stem cell transcription factor SOX2 (Gómez-López et al., 2011) and SOX9, another SOX-family member relevant for NSC self-renewal (Scott et al., 2010). Neither of these markers, clearly expressed in non-irr NSCs, were detected in most nuclei analyzed at day 7 after irr (Figures 2D, S2B, and S2C). Progressive differentiation toward the astrocytic lineage was indicated by an increasing fraction of cells positive for the astrocyte-typical membrane channel Aquaporin 4 (Yoneda et al., 2001) at day 7 post-irr (Figures 2D and S2D). Western blotting confirmed reduction of Nestin, the self-renewal-promoting transcription factors PAX6 (Gómez-López et al., 2011) and OLIG2 (Ligon et al., 2007), and upregulation of GFAP in irr NSCs (Figures 2E and S2E).

We consistently detected downregulation of *Nestin*, *SOX2*, and *PAX6* mRNA by quantitative real-time PCR in several independent irr experiments (Figure 2F). Moreover, we observed widespread reduction in expression of genes associated with pathways typical of NSC biology and self-renewal (Figure 2G): transcription factors *SOX9* and *OLIG2*, RNA-binding proteins *Musashi-1* (Okano et al., 2002) and *ARS2* (Andreu-Agullo et al., 2012), nuclear receptor *TLX* (Qu et al., 2010), and the intermediate filament *Vimentin* (Conti et al., 2005). We extended this to genome-wide analysis in irr NSCs using cDNA microarrays. Using data sets from brain-derived astrocytes (Cahoy et al., 2008) or astrocytes differentiated in vitro from NSCs by serum stimulation (Obayashi et al., 2009) as references, we observed that numerous genes upregulated or downregulated specifically during astrocytic differentiation showed a similar pattern in irr NSCs at day 7 (Figure 2H). The shift toward the expression of astrocytic markers was not associated with augmented expression of neuronal genes (detected either by microarrays or quantitative real-time PCR), even at later time points post-irr (Figure S2F). This phenotype of DNA-damage-induced differentiation became increasingly robust over time to include 14 days post-irr the expression of *S100β* (Figures S2F and S2G), a marker of more mature astrocytes (Raponi et al., 2007). Importantly, NSCs were still tripotent shortly after irr, as they could efficiently produce TUJ1-positive neurons under appropriate differentiation conditions (Figure S2H).

### DNA Damage Leads to Cytokine Secretion and *GFAP* Gene Induction through BMP2 and JAK-STAT Signaling in Senescent NSCs

Interestingly, the efficiency of GFAP induction correlated directly with the initial cell density at the time of irr and inversely with the volume of cell culture medium (Figure 2I), implying a potential role for soluble factors secreted from irr NSCs.

**Figure 3:**
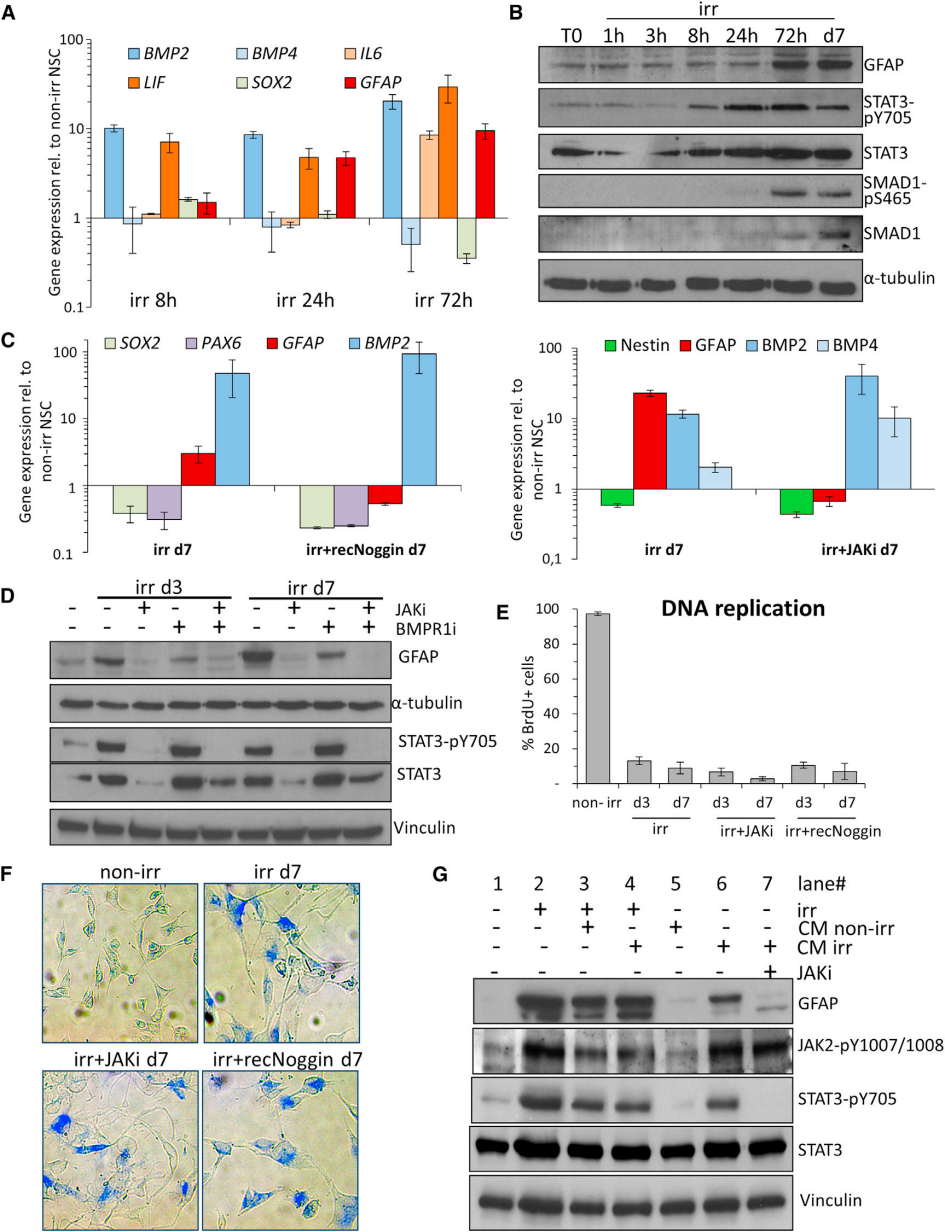
GFAP Induction in Irradiated Senescent NSC Depends on BMP2 and JAK/STAT Signaling (A) Time-course study of the cytokine expression in irr NSCs by quantitative real-time PCR. *SOX2* and *GFAP* expression reflect self-renewal and differentiation, respectively. Error bars show SD. (B) WB analysis of the time course of STAT and SMAD signaling pathway activation in irr NSCs. GFAP signal reflects the onset of differentiation. (C) Quantitative real-time PCR analysis of NSCs on day 7 post-irr. Note that continuous Noggin (left panel) or JAKi (right panel) treatment impaired *GFAP* induction, despite the ongoing expression of *BMP2* and *BMP4*. Error bars show SD. (D) WB analysis of the effects of JAK/STAT signaling inhibition (JAKi) and BMP2/4-receptor inhibition (BMPR1i) on GFAP induction. (E) BrdU incorporation-based assessment of DNA replication in irr NSCs treated continuously with JAKi or Noggin. BrdU was supplied for 24 hr prior to fixation and detected by IF and wide-field microscopy. Error bars show SD. (F) Representative wide-field microscopy images of persistent senescence-associated β-galactosidase activity in irr NSCs continuously treated with JAKi or Noggin, magnification: 20×. (G) WB analysis for the effect of conditioned medium (CM) from irr or not irr NSCs, daily collected for 3 days, and transferred onto separate irr or non-irr NSCs. Lane 7: CM from irr NSCs was supplemented with JAKi. See also Figure S3.

It is generally understood that *GFAP* gene expression during astrocytic differentiation is induced by the synergic action of cytokines of the BMP (BMP2 and 4) and interleukin (IL)-6 families (i.e., LIF), which signal, respectively, through SMAD1-SMAD4 and JAK-STAT (Fukuda et al., 2007; Nakashima et al., 1999). Other IL-6-type cytokines (CNTF, OsM, and CT-1) are also known to signal through LIF receptor (Turnley and Bartlett, 2000). Among the candidate cytokines analyzed (Table S1), we observed that *BMP2*, *BMP4*, *LIF*, and *IL-6* were consistently expressed in irr NSCs at day 7. A short time-course analysis of their expression showed a strong induction of *BMP2* and *LIF* within 8 hr of irr, preceding *GFAP* induction and *SOX2* downregulation, with *BMP4* or *IL-6* being induced only after the *GFAP* increase (Figure 3A).

BMP2 and LIF signal canonically through downstream SMAD1 and STAT3 mediators, respectively (Fukuda et al., 2007; Nakashima et al., 1999). We studied the activation of these pathways in irr NSCs by probing for the phosphorylated (activated) forms of STAT3 and SMAD1. Remarkably, the phospho-STAT3 signal increased and persisted from 8 hr after irr onward, whereas SMAD1, poorly expressed in NSCs, accumulated and became phosphorylated only 3 days post-irr (Figure 3B). Gene Ontology analysis of microarray data suggested activation of JAK-STAT pathway, whereas SMAD cascade profiling showed a very poor induction of canonical downstream genes (Figure S3A).

Next, we probed these signaling pathways using a set of specific inhibitors. The polypeptide Noggin prevents BMP2/4 signaling through the BMPR1a receptor (Lim et al., 2000). Noggin treatment of NSCs after irr prevented *GFAP* upregulation, despite robust induction of *BMP2*; a similar effect was observed upon suppression of JAK-STAT signaling with a pharmacological pan-JAK-kinase inhibitor (JAKi; Figure 3C and Figure S3B). Moreover, the use of a small-molecule inhibitor of BMPR1a kinase activity (BMPR1i) as well as JAKi both abolished GFAP induction, separately and in combination (Figure 3D). Interestingly, irr NSCs still showed STAT3 phosphorylation when BMP2/4 signaling was suppressed, and phospho-SMAD signal was detectable when JAKi was applied; this was nevertheless not sufficient to trigger GFAP induction (Figures 3D and S3B). Suppression of glial differentiation by JAKi in irr NSCs did not result in the expression of neuronal genes (Figure S3C).

Remarkably, these inhibitors did not restore the expression of self-renewal genes (Figure 3C) or that of proliferation markers (Figure S3C), or the DNA replication activity in irr NSCs (Figure 3E). Instead, cells retained the expression of cellular senescence markers (Figures 3F and S3D), thus ruling out the possibility of cellular quiescence, reported elsewhere as associated with GFAP upregulation and controlled by BMP2/4 (Mira et al., 2010; Sun et al., 2011). Consistent with the ablation of DDR-suppressing astrocytic differentiation (Schneider et al., 2012), we detected a higher residual DDR activity in JAKi-treated irr cells compared to DMSO-treated irr cells (Figures S3E and S3F).

Next, we collected conditioned medium (CM) from non-irr and irr cells and tested its effect on non-irr NSCs (Figure 3G). Strikingly, CM from irr cells, unlike that from non-irr cells, was sufficient to induce robust STAT3 phosphorylation and GFAP induction in non-irr cells Importantly, this effect was suppressed by supplementing the CM with the JAKi prior to application (Figure 3G). Thus, growth factors secreted by irr NSCs mediate their astrocytic differentiation by activating the JAK-STAT signaling pathway.

### GFAP Induction and Astrocytic Differentiation Rely on Noncanonical BMP2 Signaling through JAK-STAT

**Figure 4:**
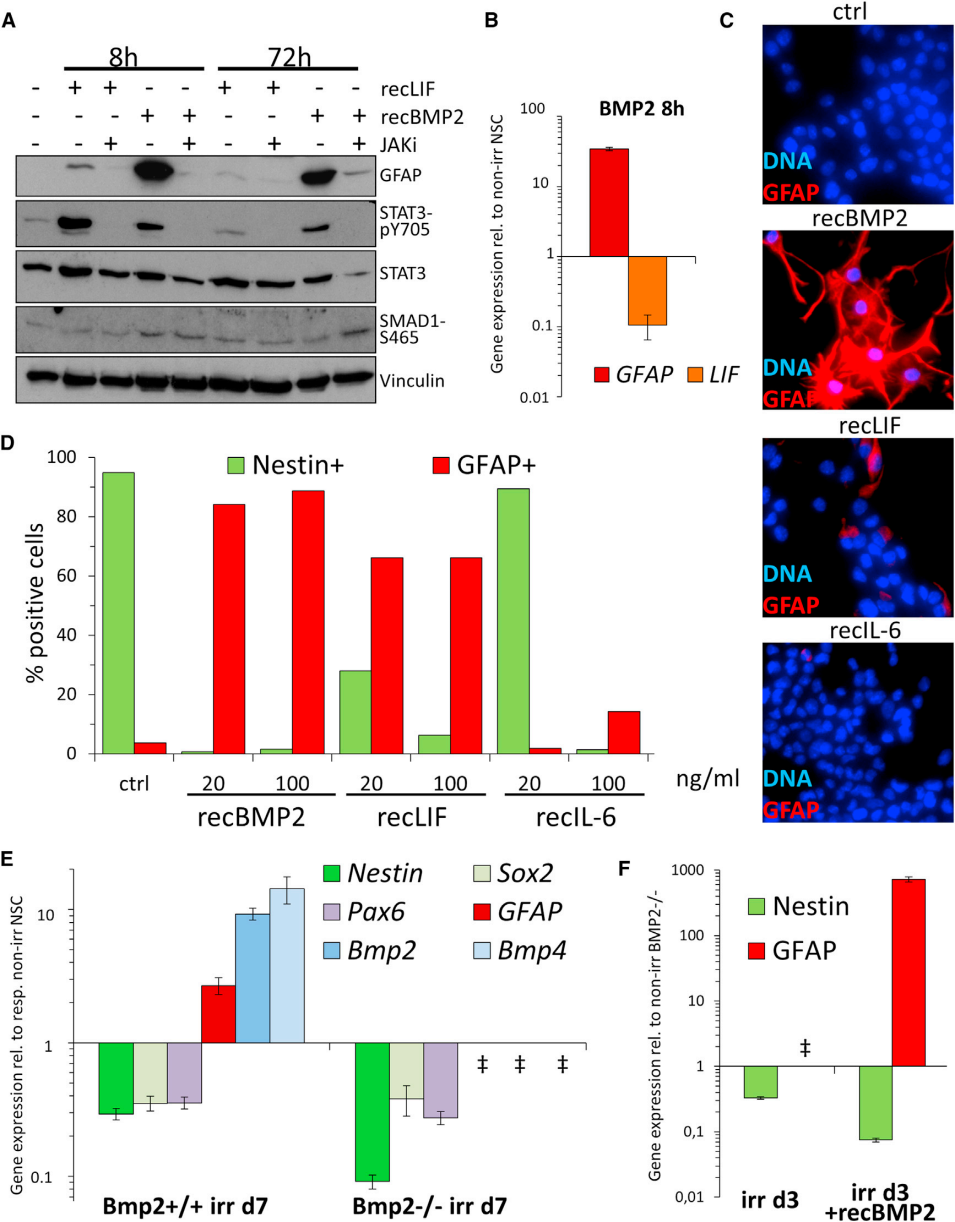
BMP2 Signals Noncanonically through JAK/STAT to Induce GFAP Independently of LIF (A) WB analysis of the effects of the cytokines LIF and BMP2 at 20 ng/ml on GFAP induction and the role of JAK/STAT signaling in non-irr NSCs. (B) Quantitative real-time PCR analysis of *GFAP* and *LIF* expression levels in NSCs exposed for 8 hr to 20 ng/ml recombinant BMP2. Error bars show SD. (C) Representative wide-field microscopy images of the effect recombinant cytokines (20 ng/ml) on NSCs after 6 days. GFAP detected by IF analysis, magnification: 40×. (D) Quantification of Nestin and GFAP-positive cells after cytokine exposure for 6 days at 20 ng/ml or 100 ng/ml. (E) Quantitative real-time PCR analysis of wild-type and isogenic *BMP2*-deficient NSCs for the expression of *BMP2/4*, NSC markers *Nestin*, *SOX2*, *PAX6*, and the astrocyte marker *GFAP*. Error bars show SD. ‡*GFAP*, *BMP2*, and *BMP4* gene expression were not detectable in irr BMP2^−/−^ NSCs. (F) Quantitative real-time PCR analysis of *BMP2*-deficient NSCs for the expression of *Nestin* and *GFAP*. Error bars show SD. ‡As in (E), *GFAP* not detectable.

NSCs upregulated *BMP2* as well as *LIF* immediately after irr (Figure 3A); however, only *BMP2* expression remained stable even 1 month after irr (Figure S2G). To investigate the individual roles of these two cytokines, we treated non-irr NSCs with either BMP2 or LIF in the presence or absence of JAKi. LIF has been reported to stimulate GFAP expression by upregulating BMP2 (Fukuda et al., 2007). Predictably, LIF activated JAK-STAT signaling and induced GFAP; both events were prevented by JAKi (Figure 4A). Surprisingly, BMP2 not only proved a more potent GFAP inducer than LIF, that alone was sufficient to activate JAK-STAT signaling, both effects also were fully abolished by JAKi (Figure 4A). Importantly, BMP2 treatment did not stimulate transcriptional induction of *LIF* (Figure 4B). Moreover, whereas BMP2 exposure resulted in astrocyte-typical morphology change in NSCs and profound GFAP upregulation, such effects were much less pronounced in LIF-treated and completely absent in IL-6-treated NSCs (Figure 4C). At 20 ng/ml, about 25% of LIF-treated NSCs and nearly all IL-6-treated cells were Nestin positive, whereas virtually all BMP2-exposed cells ceased expressing Nestin (Figure 4D). IL-6 reduced Nestin only at very high concentrations (100 ng/ml).

Finally, we took advantage of wild-type and isogenic *BMP2*-knockout murine ES cells to derive NSCs through established methods (Conti et al., 2005; Ying et al., 2003). Although irr wild-type NSCs downregulated stem cell markers *Nestin*, *SOX2*, and *PAX6* and upregulated *GFAP*, we could not detect any *GFAP* gene expression even by sensitive quantitative real-time PCR techniques in irr *BMP2*^*−/−*^ cells, despite downregulated stem cell markers (Figure 4E). Interestingly, *BMP4* was also undetectable in *BMP2*^*−/−*^ cells (Figure 4E), indicating that its expression is controlled by BMP2, as previously suggested (Castranio and Mishina, 2009). Yet irr *BMP2*^*−/−*^ NSCs proved to be fully proficient in inducing *GFAP* when exposed to recombinant BMP2 (Figure 4F).

Thus, BMP2 can signal noncanonically through JAK-STAT and induce *GFAP* expression independently from LIF or other IL-6-type cytokines.

### DNA-Damage-Induced Differentiation Requires ATM and Is Opposed by p53

**Figure 5:**
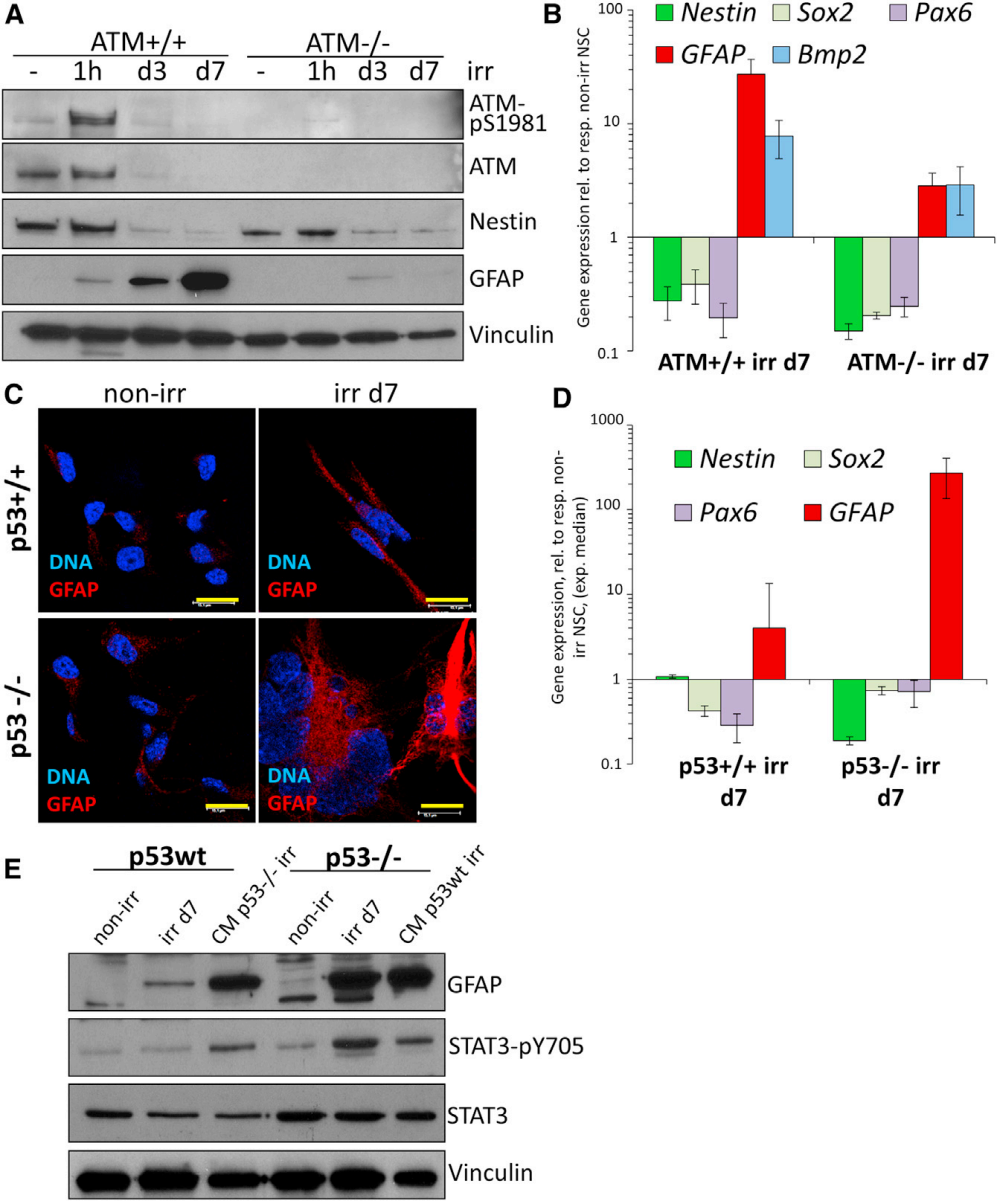
GFAP Induction Is Impaired in ATM-Deficient Irradiated NSCs, whereas p53 Deficiency Promotes DNA-Damage-Induced Differentiation through Increased Cytokine Secretion (A) WB analysis for Nestin and GFAP in irr wild-type and isogenic ATM-deficient NSCs. Membrane was also probed for phospho- and total ATM protein. (B) Quantitative real-time PCR analysis of wild-type and isogenic ATM-deficient NSCs for the expression of *BMP2*, *Nestin*, *SOX2*, *PAX6*, and *GFAP*. Error bars show SD. (C) Representative confocal images of wild-type and isogenic p53^−/−^ NSCs, GFAP detected by IF analysis. Bar: 15 μm. (D) Median values of two or more experiments of wild-type and isogenic p53^−/−^ NSCs, analyzed by quantitative real-time PCR for the expression of *Nestin*, *SOX2*, *PAX6*, and *GFAP*. Error bars show SEM. (E) WB analysis for the effect of conditioned medium (CM) from irr p53^−/−^ NSCs transferred on irr wild-types (promoting STAT3 phosphorylation and GFAP induction) and vice versa. See also Figure S4.

Previous studies established a mechanistic link between the DNA-damage-induced permanent proliferative arrest and cytokine secretion, described as senescence-associated secretory phenotype or SASP, as secretion of IL-6/8 requires ATM and is opposed by p53 (Coppé et al., 2008; Rodier et al., 2009). We investigated these molecular pathways in the context of DNA-damage-induced differentiation. First, we employed a previously characterized strain of *ATM*^*−/−*^ ES cells to generate *ATM*^*−/−*^ NSCs. Upon irr, both wild-type and *ATM*^*−/−*^ NSCs arrested proliferation (Figure S4A) and downregulated stem cell markers *Nestin*, *SOX2*, and *PAX6*, but *ATM*^*−/−*^ cells were less efficient in increasing *BMP2* and *GFAP* levels (Figures 5A and 5B).

p53 is a central downstream effector of the DDR cascade (Jackson and Bartek, 2009) and has been reported to regulate self-renewal and proliferation in NSCs (Meletis et al., 2006) and suppress SASP (Coppé et al., 2008). We used p53-deficient ES cells to derive *p53*^*−/−*^ NSCs. When irradiated, these cells exited the cell cycle (Figure S4B) and underwent irr-induced glial differentiation even more readily than isogenic wild-type cells, as suggested by a significantly stronger induction of GFAP (Figures 5C, 5D, and S4C). We next asked whether this was the consequence of stronger cytokine secretion: we irradiated p53-deficient and isogenic wild-type cells and transferred CM from irr *p53*^*−/−*^ cells onto irr isogenic wild-type NSCs. We observed a much stronger induction of phospho-STAT3 and GFAP in CM-treated irr wild-type NSCs than in CM-untreated cells (Figure 5F).

### DNA Damage In Vivo Leads to Astrocytic Differentiation of NSCs in the SVZ

**Figure 6:**
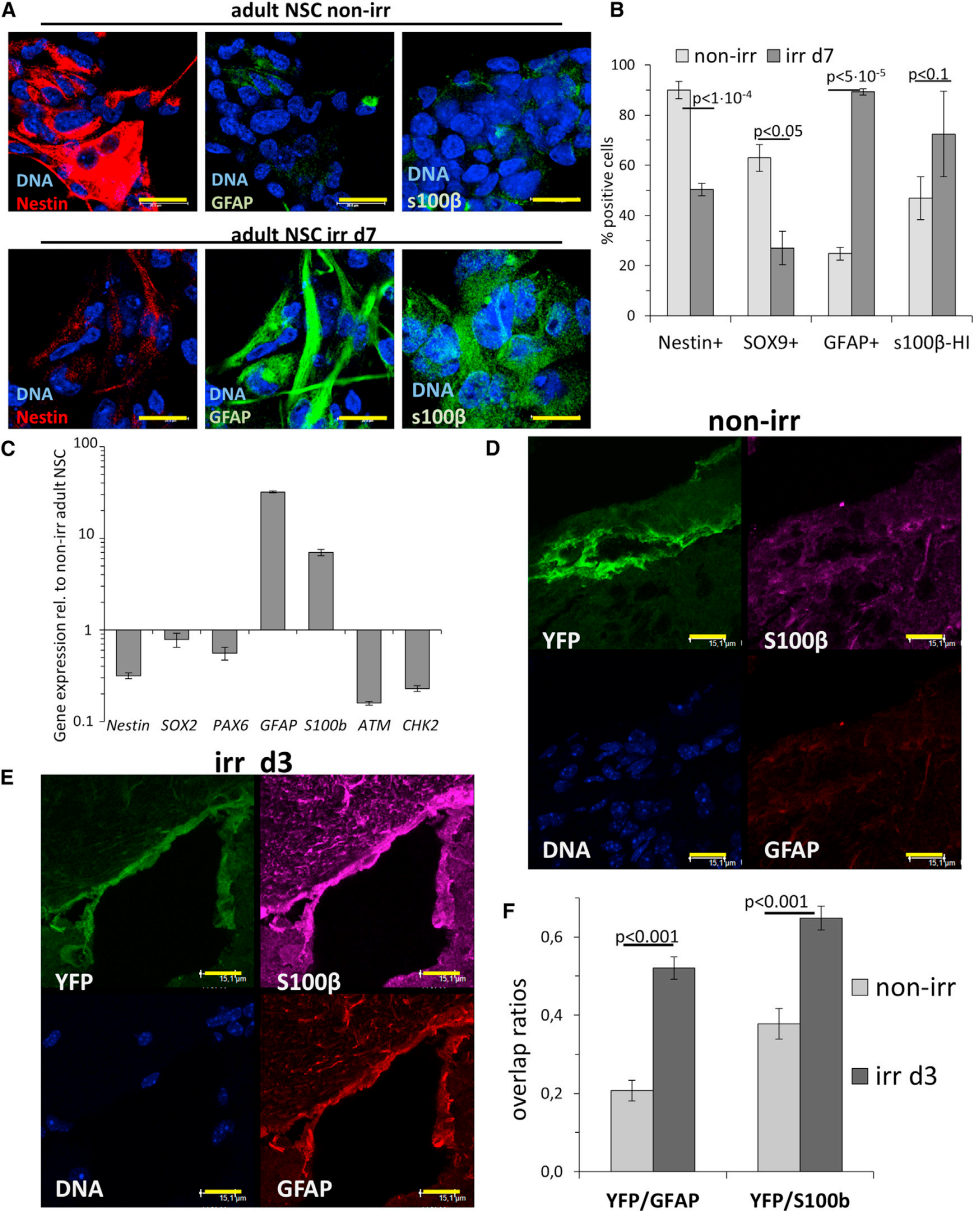
NSCs from Adult Forebrain Undergo Astrocytic Differentiation upon Irradiation In Vitro as Well as in a Mouse Model of Cell-Fate Tracing (A) Representative confocal images of non-irr adult NSCs and cells at day 7 post-irr; Nestin and astrocyte markers GFAP and S100β were detected by IF analysis. Bar: 20 μm. (B) Quantification of adult NSCs at day 7 post-irr for the Nestin and GFAP expression, nuclear SOX9 signal, and the highly S100β-positive cells (s100β-HI) as analyzed by IF and wide-field microscopy. (C) Quantitative real-time PCR analysis of adult NSCs on day 7 post-irr for the expression of self-renewal (*Nestin*, *SOX2*, *PAX6*) and astrocyte markers (*GFAP*, *S100β*), and DDR genes (*ATM*, *CHK2*). Error bars show SD. (D) Mice were treated with tamoxifen to label SOX2 expressing cells, mock irradiated, and sacrificed 3 days later. Brain sections containing the SVZ were stained by triple IF with an anti-YFP antibody in order to detect labeled cells and antibodies against astrocyte markers GFAP and S100β to address differentiation status. A collapsed confocal microscopy z stack for each channel is shown. Bar: 15 μm. The merged collapsed z stack of all channels is provided in Figure S5D. (E) Mice were treated as above, but subjected to cranial irradiation. Brain sections containing the SVZ were analyzed as above. Bar: 15 μm. The merged collapsed z stacks of all channels is provided in Figure S5E. (F) Three non-irr and irr brains each from two irradiation experiments were analyzed to obtain approximately ten confocal z stack series from several physical sections of each brain’s SVZ (<30 z stacks for each condition in total). The colocalization ratio of the YFP signal with astrocyte markers GFAP and S100β was calculated for each layer of the z stack as the Mander’s coefficient of YFP overlap with GFAP or S100β; median values are shown. p values were calculated by Mann-Whitney rank sum test. Error bars: SEM. See also Figure S5.

NSCs used in this study so far were derived from ES cells and resemble more closely embryonal radial glia than type B cells residing in adult brain (Conti et al., 2005; Doetsch, 2003; Pollard et al., 2006). Thus, we next employed cell lines derived from adult mouse forebrain (Conti et al., 2005; Pollard et al., 2006). Consistent with our results with ES-derived NSCs, nearly all adult NSCs entered cellular senescence upon irr (Figures S5A and S5B). Compared to ES cell-derived NSCs, these cells also displayed a striking increase in GFAP expression upon irr (Figures 6A and S5C), likely reflecting the more primed state of the *GFAP* promoter (Doetsch, 2003). Astroglial differentiation of adult NSCs was also demonstrated by upregulation of *S100β* mRNA and protein (Figures 6B and 6C) and a strong reduction of Nestin protein and mRNA, as well as other stem cell markers (SOX2, SOX9, PAX6) and DDR genes (*ATM*, *CHK2*) (Figures 6A–6C).

Next, we extended our study to living animals and took advantage of a mouse strain in which NSCs can be labeled in vivo noninvasively and permanently (Favaro et al., 2009). These mice express *Cre* endonuclease fused to estrogen receptor (ERT2), under a *SOX2* 5′ telencephalic enhancer/promoter. The animals were crossed with a strain carrying R26-eYFP-fLox-STOP transgene (Srinivas et al., 2001). In this way, treatment with tamoxifen allowed permanent labeling of *SOX2-*expressing cells with YFP, which is retained independently of *SOX2* expression afterward.

We exposed mice with in-vivo-labeled SOX2-expressing NSCs to cranial irradiation with 10 Gy (or mock irradiation), sacrificed them on day 3 after irr, and analyzed their brains by immunofluorescence and confocal microscopy. In mock-irradiated (non-irr) animals, YFP-positive cells in the SVZ rarely expressed detectable GFAP or S100β (Figures 6D and S5D). Strikingly, in irr brains, YFP-expressing cells were often positive for both GFAP and S100β as detected by triple immunofluorescence, indicating astrocytic differentiation of SOX2-expressing cells in SVZ within 3 days after irr (Figures 6E and S5E). Importantly, as an internal control, immunostaining for YFP showed similar intensities in both irr and non-irr brains.

Because the astrocyte markers GFAP and S100β are both detectable in the cytoplasm, we could calculate the ratio with which the cytoplasmatic YFP signal overlapped quantitatively with those, in experimental animal triplicates. Quiescent radial glia in the SVZ (type B cells) express GFAP (Bonfanti and Peretto, 2007), and indeed we detected some overlap of YFP and GFAP signals in non-irr brains (∼20%). Moreover, due to prolonged tamoxifen treatment (10 days), a degree of homeostatic astroglial differentiation of previously labeled NSCs was expected. Nevertheless, in irr brains at day 3 we measured a statistically significant ∼2-fold increase in the overlap of YFP signal with GFAP signal as well as with S100β signal, when compared to non-irr controls (Figure 6F).

### DNA Damage in Glioblastoma Stem Cells In Vitro and in Mouse Glioblastoma Model In Vivo Leads to Astrocytic Differentiation and Loss of Tumorigenic Potential

**Figure 7:**
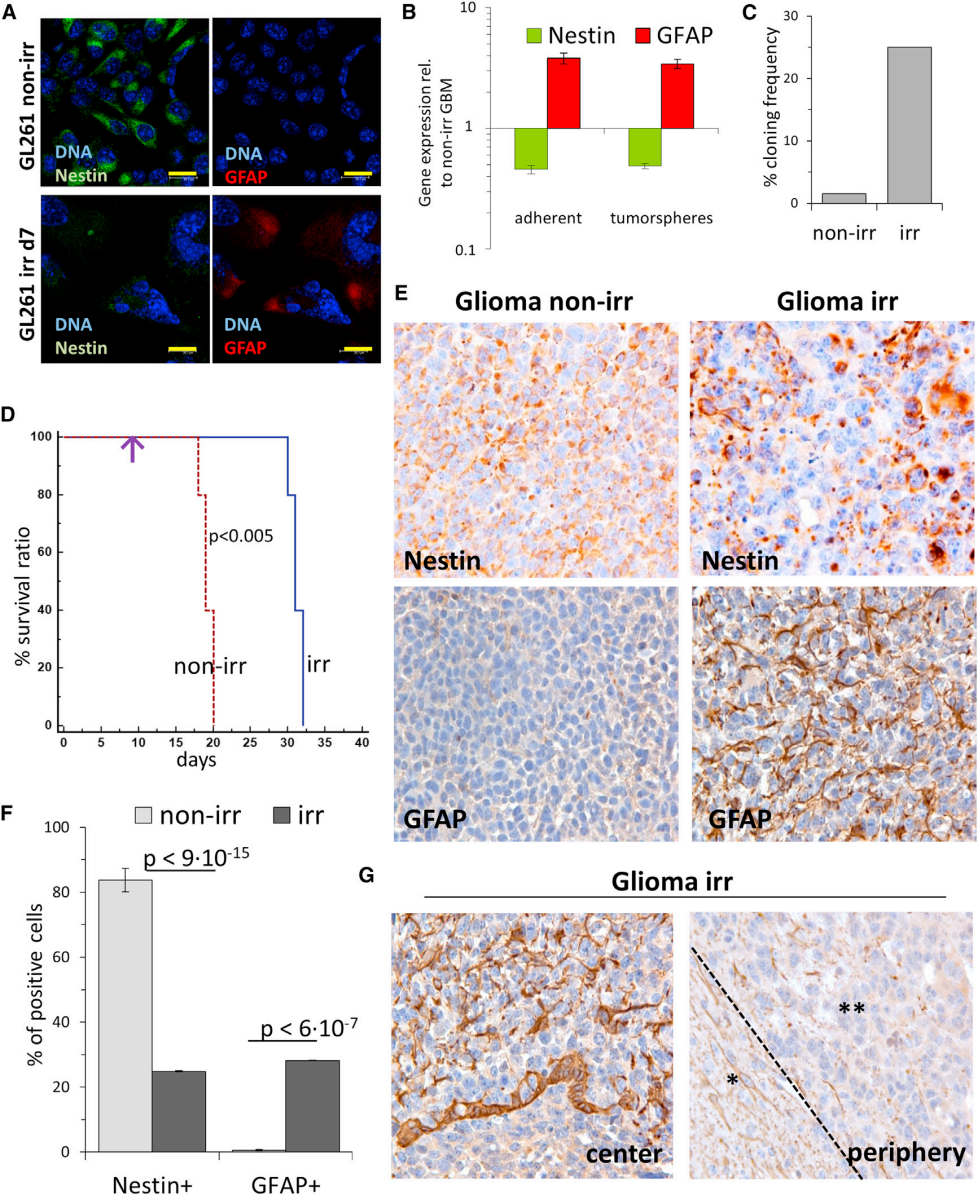
Murine Glioblastoma Stem Cells Undergo Astrocytic Differentiation In Vitro and in a Mouse In Vivo Xenograft Model (A) Representative confocal images of murine GBM cell line GL261-CSC, irradiated in adherent conditions, Nestin and GFAP detected by IF analysis. Bar: 20 μm. (B) Quantitative real-time PCR analysis (TaqMan assay) of GL261-CSC grown in serum-free tumorsphere and adherent cultures on day 3 post-irr for the expression of *Nestin* and *GFAP*. Error bars show SD. (C) In vitro cloning analysis performed by serial dilution in 96-well plates on non-irr and irr GL261-CSC. (D) GBM tumors were induced in mice by injection of 10^5^ GL261 cells. Radiation therapy was applied at day 10 (arrow). Kaplan-Meier curves (five animals each) show significantly prolonged survival of GBM-tumor-bearing mice after cranial irr. (E) Representative immunohistochemistry (IHC) analysis of Nestin and GFAP expression in GBMs from tumor-bearing animals, sacrificed 10 days after radiation therapy. Upper panel: large areas of GBM tumors became negative for Nestin after irr. Lower panel: whereas non-irr GBM are generally GFAP negative, radiation therapy results in a tremendous increase in the GFAP signal. Magnification: 40×. (F) Quantification of Nestin and GFAP-positive cells in the GBM tumor mass of non-irr mice or those subjected to radiation therapy. Two animals were studied for each condition. Error bars show SD. (G) Representative IHC analysis of GFAP expression in irr GBM tumors at day 30 (=day 20 after radiation therapy at day 10). Note that in advanced tumors the differentiation marker GFAP could was prominent only in the central glioma mass (top panel), but not in the expanding tumor periphery (bottom panel; ^∗^healthy tissue, ^∗∗^tumor, separated by dashed line). Magnification: 40×. See also Figure S6.

Finally, we extended our study to brain cancer cells, which share key functional characteristics with untransformed NSCs, such as self-renewal and the gene expression signature (Nicolis, 2007). We exposed to 10 Gy irr a murine glioblastoma (GBM) cancer stem cell line (GL261-CSC), which carries point mutations in *K-Ras* and *p53* (Szatmári et al., 2006), expresses Nestin but not GFAP, and can be grown as adherent cultures as well as tumorspheres (Pellegatta et al., 2006). Here also, irradiation triggered downregulation of Nestin and induction of GFAP, both in tumorspheres and in adherent cells (Figures 7A, 7B, S6A, and S6B). Although the clonal frequency of non-irr GBM cells was 25%, after irr it dropped to only 1.5% of the total number of cells plated (Figure 7C). Correspondingly, key proliferation genes were downregulated in irr GBM cells (Figure S6C).

GL261-CSCs are widely used to simulate human GBMs and initiate aggressive and lethal brain tumors in rodent assays (Jacobs et al., 2011). We tested the in vivo tumorigenicity of irr versus non-irr GBM cells by grafting them into mouse brains. Three days after in vitro irr of GBM cells, ten animals each were injected into the nucleus caudatum with 10^3^, 10^4^, or 10^5^ irr GL261-CSC or with equal numbers of non-irr cells. Non-irr GBM cells formed aggressive tumors and proved lethal to all host animals in about 20–40 days (Figure S6D; Table S2). By contrast, when 10^3^ irr GBM cells were injected, no tumor-induced mortality was observed during the entire observation period of 100 days, whereas 10^4^ or 10^5^ injected irr cells led to markedly reduced mortality (Figure S6D; Table S3).

Finally, we probed DNA-damage-induced differentiation in murine glioblastoma in vivo by injecting 10^5^ non-irr GL261-GLS and 10 days later exposing the glioma-bearing mice to focused cranial irradiation of 10 Gy. Radiation therapy led to a significant increase in survival as compared to mock-irradiated glioma-bearing mice (Figure 7D). We examined irr and non-irr gliomas from mice sacrificed at two time points. Ten days after irr, the tumor mass was small and localized near the injection site with necrotic areas, whereas the non-irr glioma was much larger and infiltrated the contralateral hemisphere, showing high cellularity (Figure S6E). The majority of GBM cells in non-irr tumor reflected their stem cell characteristics by a strong Nestin signal and a low GFAP presence. Upon irr, the majority of GBM cells lost their Nestin expression, whereas a large fraction of GBM cells near central tumor mass strongly upregulated the astrocytic differentiation marker GFAP (Figures 7E and 7F).

Gliomas from mice sacrificed 20 days after irr, however, displayed Nestin-positive cells preferentially located at tumor borders (34.6% ± 9.1% in the periphery and 10.6% ± 1.3% in the center, Figure S6F). Correspondingly, GFAP-positive cells were located in the central tumor mass and not in the periphery (15.6% ± 1.8% center versus 1.2 ± 1.2, periphery; (Figures 7G and S6G), where the tumor did progress. In summary, tumor growth requires a stem-like state of glioblastoma cells, and radiation therapy induces their differentiation and decreases their oncogenic potential.

## Discussion

This study demonstrates that DNA damage in NSCs leads to cellular senescence, depriving them of their self-renewal potential and promoting astrocytic differentiation. This process is niche independent; i.e., it occurs even under self-renewal-promoting culture conditions and relies on the cell-autologous DNA-damage-induced secretion of soluble factors. Our results also highlight a noncanonical BMP2 signaling pathway through JAK-STAT, which is responsible for promoting astrocytic differentiation of senescent cells. Moreover, our conclusions apply both in vitro and in vivo, including adult brain NSCs and GBM stem cells.

Terminal differentiation of stem and progenitor cells is defined by an irreversible cell-cycle arrest, loss of expression of stem/progenitor cell markers, and upregulation of differentiation-associated genes. We observed this in both ES-derived and adult forebrain NSCs after irr. Moreover, we also observed loss of DDR signaling and DDR gene expression in irr NSCs, which is consistent with their differentiation toward the astrocytic lineage (Schneider et al., 2012). The differentiation bias of irr NSCs toward astrocytes may be explained by their glial nature (Doetsch, 2003). Indeed, NSCs sustaining mitochondrial DNA damage were reported to be more prone to astroglial fate when stimulated to differentiate (Wang et al., 2011).

In our model, DNA damage forces cells into cellular senescence, whereas ATM-dependent and p53-antagonized cytokine secretion activates BMP2/JAK-STAT signaling and stimulates the differentiation process in a progressive feed-forward manner. This senescent state is very different from the GFAP-associated quiescence described elsewhere (Mira et al., 2010; Sun et al., 2011), because quiescent NSCs are characterized by retention of their self-renewal profile. Moreover, this NSC-specific cellular senescence takes place in the absence of persistent DDR signaling, which is commonly required for senescence maintenance in non-stem cell types (d’Adda di Fagagna, 2008; Jackson and Bartek, 2009). Hence, these cellular senescence and ablation of self-renewal are likely to involve epigenetic mechanisms that persist after initial DNA-damage-induced cues.

Telomere-attrition-induced DNA damage in hematopoietic stem cells activates STAT3 and, in turn, BATF in a G-CSF-dependent manner, leading to their differentiation (Wang et al., 2012). Although our microarray data do not indicate this particular signaling activity in irr NSCs, STAT3 seems an important differentiation pathway as suggested by this and other studies (Fukuda et al., 2007; Lee et al., 2010). BMP2 and BMP4, which bind to the same receptor BMPR1, were shown to induce differentiation of glioblastoma-initiating cells (Piccirillo et al., 2006). In the nervous system, BMP2/4 is thought to act in concert with LIF (or another relevant IL-6 family member like CNTF), signaling through SMAD1 and JAK2-STAT3, respectively, to induce GFAP (Fukuda et al., 2007; Nakashima et al., 1999). Others have suggested that BMP2/4 may directly activate STAT3 signaling (Jeanpierre et al., 2008), and BMP4 alone was reported to induce GFAP (Obayashi et al., 2009). Our study provides evidence that BMP2 can activate JAK-STAT signaling and induce GFAP independently of LIF, whereas still requiring binding to its receptor and stimulating its kinase activity.

We also demonstrate here that radiation therapy forces GBM cells to lose self-renewal and commit to terminal differentiation. Whether tumor progression and mortality are caused by the escape of some GBM cells from irr-induced differentiation and the ensuing expansion of clones retaining stem cell properties remains to be defined. Future therapeutic strategies might combine radiation therapy and treatment with differentiation-promoting cytokines to ensure the permanent ablation of self-renewal in GBM.

Previous studies have indicated that NSCs in irradiated rodent brain activate cell-cycle checkpoints and lose their neurogenic capacity (Acharya et al., 2010; Monje et al., 2002). Other reports extended the concept of DNA-damage-induced differentiation to other types of somatic stem cells in vivo, such as hair bulge melanocyte stem cells (Inomata et al., 2009) and hematopoietic stem cells (Wang et al., 2012). Together with our findings, these convergent lines of evidence suggest that DNA-damage-induced differentiation may have been selected during evolution to disarm the oncogenic potential of damaged stem cells without the side effects associated with their physical elimination.

## Experimental Procedures

### ES-Derived Neural Stem Cell Lines

NSC culture and derivation from murine embryonic stem cells (ESCs) of various genetic background, based on protocols established by A. Smith’s laboratory (Conti et al., 2005; Ying et al., 2003) are described in detail in Supplemental Experimental Procedures. Predominantly, NSCs derived from E14Tg2a ES background (Burgold et al., 2008) were used. Gene-deficient ESC lines were used together with isogenic wild-types to derive gene-deficient NSCs and kindly provided as follows: BMP2^−/−^, Trisha Castranio and Yuji Mishina (NIEHS-NIH, USA and U. of Michigan, respectively); ATM^−/−^, Yang Xu (UCSD); and p53^−/−^, Jean-Christophe Marine (VIB, Belgium). References for the original ES cell strains are available in Supplemental Experimental Procedures.

### Cell Treatments

X-ray irradiation of cells was performed in a Faxitron RX-650 device at ∼2 Gy/min for 5 min (total of 10 Gy). Cells were not passaged after irr and medium change was performed on day 1 after irr and then every other day. BrdU was applied at 3.3 μM for 24 hr; JAKi I (Calbiochem) and LDN193189 (BMPR1 inhibitor; Axon Medchem) at 1 μM, with DMSO as control. Recombinant murine Noggin, LIF, IL-6, and human BMP2 (Prospec) were applied at 200 ng/ml (Noggin) and 20 ng/ml (unless stated otherwise). CM supernatants were collected daily, filtered with 0.45 μm filters and supplemented with one-third of fresh medium. In vitro cloning dilution assays on GL261-CSC were performed by dissociation of 10 Gy irr tumorspheres into single cells, plated after serial dilution as 1 cell/well in 96-well plates (n = 10/condition) and scored after 10 days for clonally derived secondary spheres.

### Animal Treatments

For in vivo cell-fate tracing, SOX2-CreERT2 mice (Favaro et al., 2009) were crossed onto R26::loxP-stop-loxP::YFP background (Srinivas et al., 2001), treated with tamoxifen, irradiated with a RADGIL irradiator, sacrificed 3 days later, and processed as described in detail in Supplemental Experimental Procedures.

For in vivo irradiation, C57BL/6N mice received brain injection of 10^5^ GL261 cells, 10 days after tumor implantation mice were cranially irradiated using a 6 MeV Varian linear accelerator at a dose of 10 Gy. The eyes were covered using a protective lead band. Ten animals each were evaluated for survival analysis; two of each group for histological analysis when moribund. Two mice (n = 1/group) were sacrificed 20 days after tumor implantation to evaluate the glioma engraftment.

### Microscopy Analyses

Details of microscopy analyses are provided in Supplemental Experimental Procedures. Cells were fixed and used for senescence assays or stained with primary antibodies for immunofluorescence. Brain sections were treated for immunofluorescence (IF) with 50 mM NH_4_Cl for epitope recovery and permeabilized/blocked with 0.2% Triton X-100 and 1% BSA in PBS. DNA was stained with DAPI (Sigma-Aldrich). For immunohistochemistry (IHC), paraffin-embedded tumor sections were probed and acquired using a Leica MDLB microscope. The percentages of Nestin and GFAP cells were calculated in in triplicate by two observers (F.P. and S.P.), indiscriminately for tumor center and periphery.

### Immunoblotting

Cells were lysed and analyzed by western blotting using primary antibodies as described in detail in Supplemental Experimental Procedures. Membrane equal loading was assessed with probing for α-tubulin or vinculin.

### Gene Expression Analysis

RNA extraction and SYBR-Green-based real-time quantitative PCR gene expression analyses were performed using primers designed with Roche UniversalProbe Library online software against *Mus musculus* as described in detail in Supplemental Experimental Procedures. In all experiments, *β2-microglobulin* (*B2M*) was used as housekeeping gene.

### Microarray Analysis

Irradiation experiments on NSCs were performed in a quadruplicate, four of each control (C1–4), and day 7 post-irr (I1-4) RNA extractions were performed as above. Labeled complementary RNA was hybridized on Affymetrix GeneChip Mouse Genome 430 2.0 Arrays, containing 45,101 probe sets corresponding to over 39,000 transcripts. Analyses and calculations were performed as described in detail in Supplemental Experimental Procedures.

### Flow Cytometry

Cells were stained live in suspension on ice with SSEA-1 antibody (#3063-25 BioVision) and then with Alexa-Fluor-488-labeled secondary antibody (Invitrogen). Cells stained with secondary antibodies only were used as negative controls. Immediately after staining, data were acquired and quantified by fluorescence-activated cell sorting on Becton Dickinson FACScalibur.

## Accession Numbers

The microarray data discussed here have been deposited in the GEO database with accession number GSE38031.

## Supplemental Information

Document S1. Supplemental Experimental Procedures, Figures S1–S6, and Tables S1–S4
