# Supplementary material for: DNA Damage in Mammalian Neural Stem Cells Leads to Astrocytic Differentiation Mediated by BMP2 Signaling through JAK-STAT
Source: Stem Cell Reports. 2013 Jul 25;1(2):123–38. doi: 10.1016/j.stemcr.2013.06.004 (PMC3757751; doi:10.1016/j.stemcr.2013.06.004)
Supplement: Document S1. Supplemental Experimental Procedures, Figures S1–S6, and Tables S1–S4 [file mmc1.pdf]

Stem Cell Reports, Volume *1*

Supplemental Information

DNA Damage in Mammalian Neural Stem Cells Leads to Astrocytic Differentiation Mediated by BMP2 Signaling through JAK-STAT

Leonid Schneider, Serena Pellegatta, Rebecca Favaro, Federica Pisati, Paola Roncaglia, Giuseppe Testa, Silvia K. Nicolis, Gaetano Finocchiaro, and Fabrizio d'Adda di Fagagna

Inventory of Supplemental Information

Supplemental Data

Fig. S1 related to **Fig. 1:** simplified scheme of the DDR signaling cascade, additional data on cell cycle arrest, DDR and DNA repair in irradiated NSC.

Fig. S2 related to **Fig. 2**: additional data on the nature of astrocytic differentiation in irradiated NSC (also fractioned irradiation).

Fig. S3 related to **Fig. 3:** additional data on the functional role of BMP2 and JAK-STAT signaling on differentiation and senescence in irradiated NSC

Fig. S4 related to **Fig. 5:** additional data on the role of ATM and p53 in irradiated NSC

Fig. S5 related to **Fig. 6:** additional data on irradiated adult NSC *in vitro* and *in vivo*.

Fig. S6 related to **Fig. 7:** additional data on irradiated glioblastoma cells (GL261-GSC) and tumors *in vitro* and *in vivo*.

Supplemental Figure Legends

Supplemental Tables

Table S 1: Effects of candidate JAK-STAT signaling cytokines on the astrocytic differentiation of NSC.

Table S 2: Survival ratios of mice after cranial injection of non-irradiated GL261-GCS.

Table S 3: Tumor incidence and mortality in mice after cranial injection of irradiated GL261-GCS.

Table S 4: Statistics checklist for main and supplemental data

Supplemental Experimental Procedures

Supplemental References

Figure S1

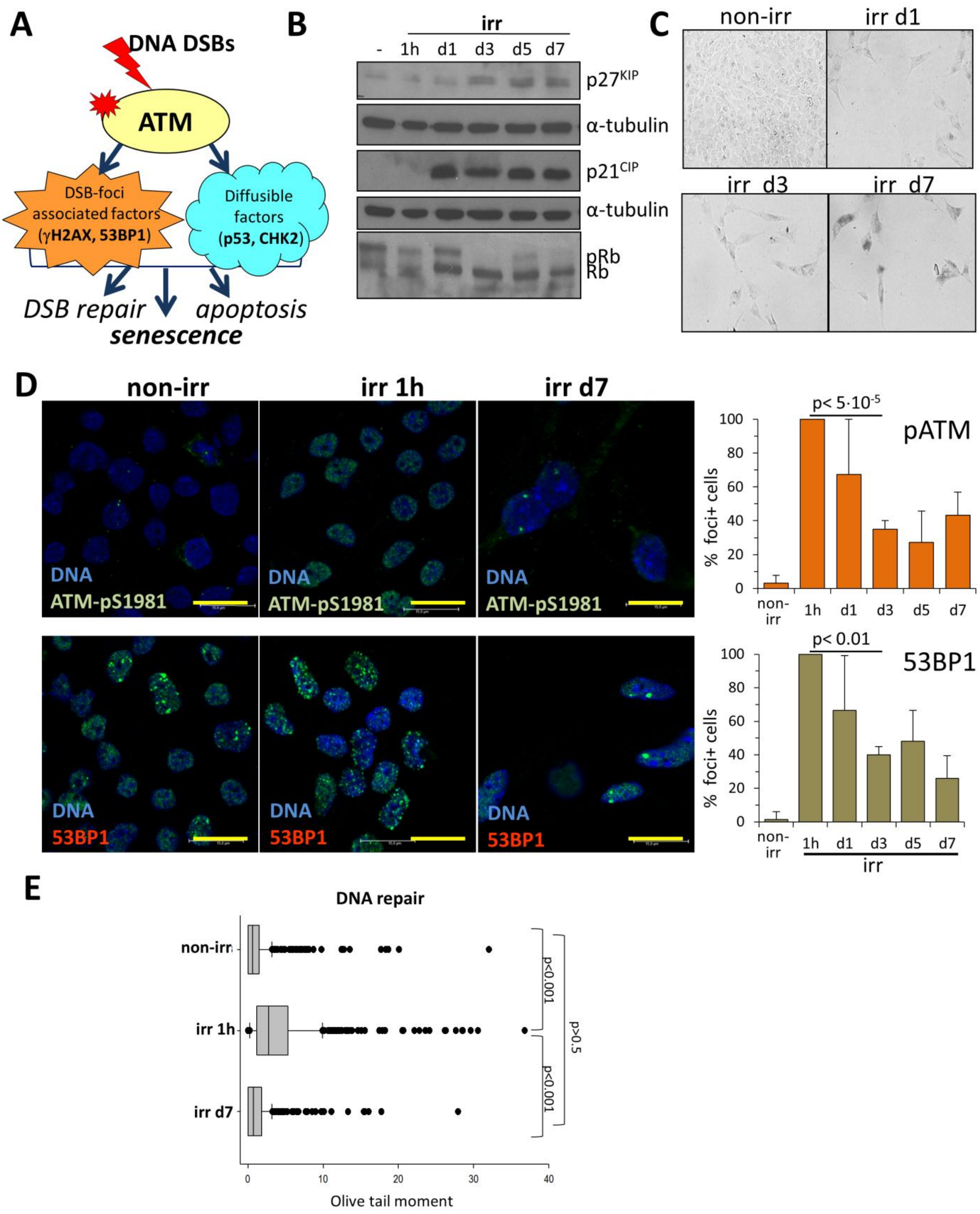
A
DNA DSBs
ATM
DSB-foci associated factors (γH2AX, 53BP1)
Diffusible factors (p53, CHK2)
DSB repair
apoptosis
senescence
B
irr
- 1h d1 d3 d5 d7
p27KIP
α-tubulin
p21CIP
α-tubulin
pRb
Rb
C
non-irr
irr d1
irr d3
irr d7
D
non-irr
irr 1h
irr d7
DNA
ATM-pS1981
53BP1
p< 5·10-5
pATM
p< 0.01
% foci+ cells
non-irr
1h d1 d3 d5 d7
irr
E
DNA repair
non-irr
irr 1h
irr d7
p<0.001
p>0.5
Olive tail moment

Figure S2

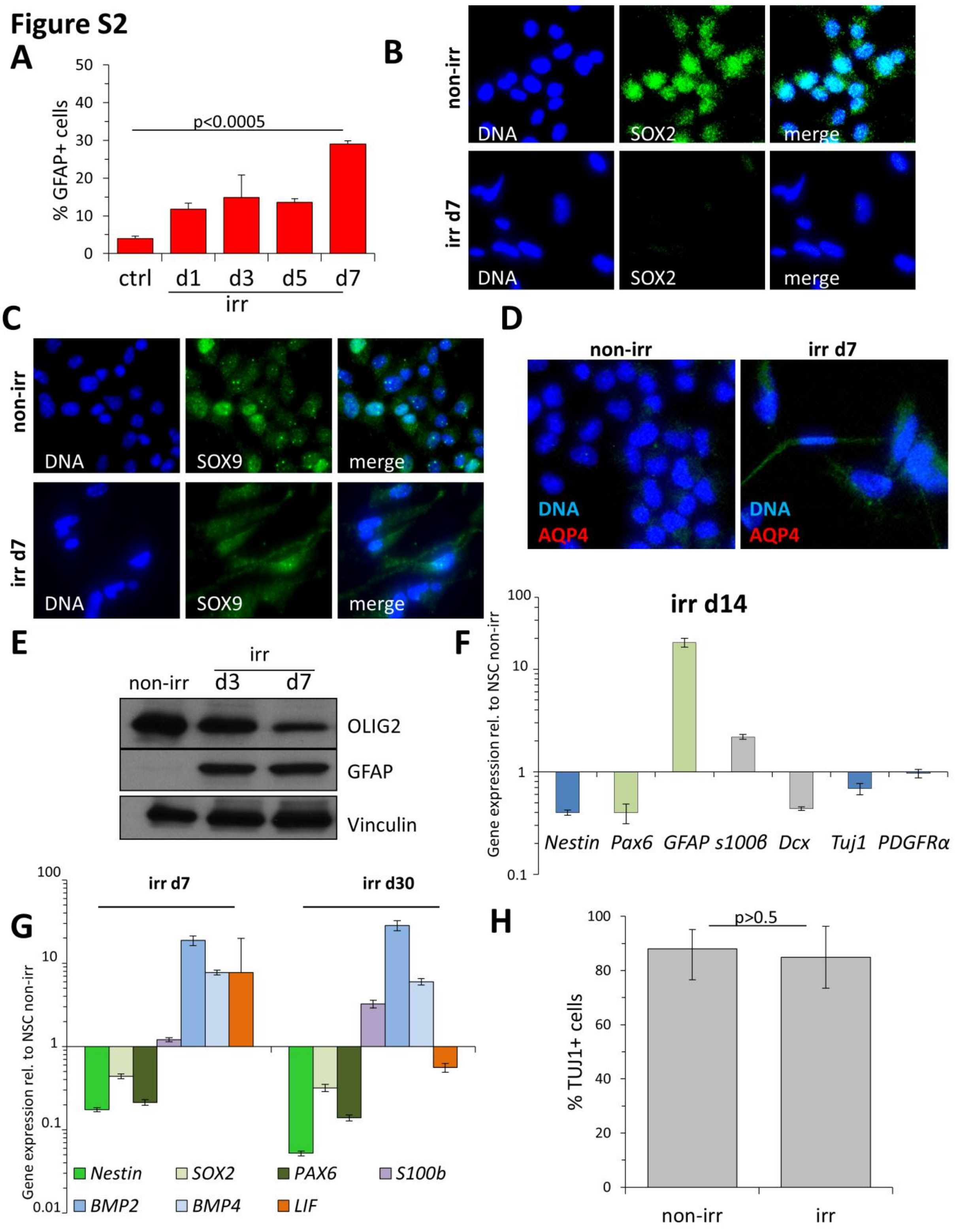
A
% GFAP+ cells
50
40
30
20
10
0
p<0.0005
ctrl
d1
d3
d5
d7
irr
B
non-irr
irr d7
DNA
SOX2
merge
C
non-irr
irr d7
DNA
SOX9
merge
D
non-irr
irr d7
DNA
AQP4
E
irr
non-irr
d3
d7
OLIG2
GFAP
Vinculin
F
irr d14
Gene expression rel. to NSC non-irr
100
10
1
0.1
Nestin
Pax6
GFAP
s100β
Dcx
Tuj1
PDGFRα
G
irr d7
irr d30
Gene expression rel. to NSC non-irr
100
10
1
0.1
0.01
Nestin
SOX2
PAX6
S100b
BMP2
BMP4
LIF
H
% TUJ1+ cells
100
80
60
40
20
0
p>0.5
non-irr
irr

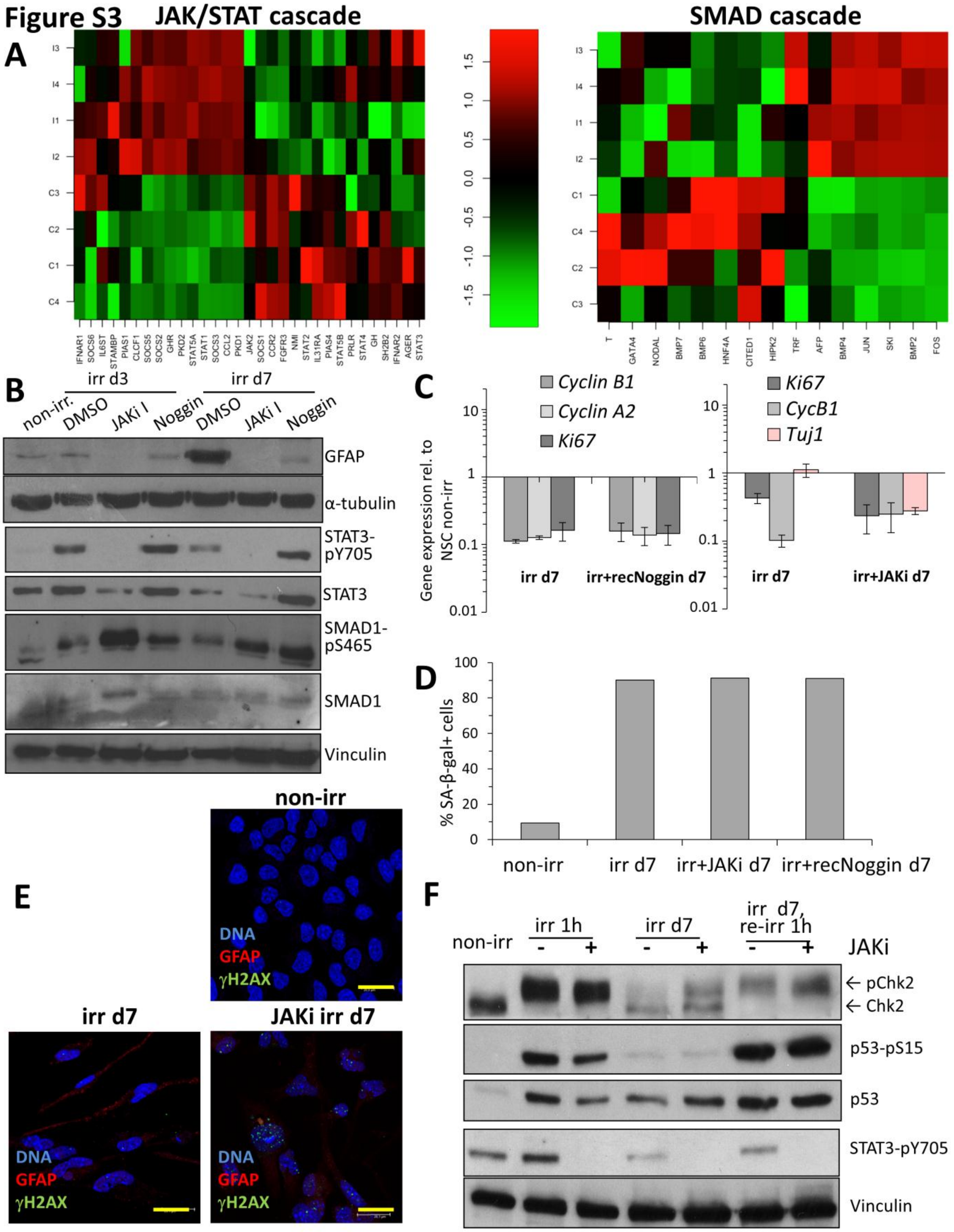
Figure S3
A
JAK/STAT cascade
I3
I4
I1
I2
C3
C2
C1
C4
IFNAR1
SOCS6
IL6ST
STAMBP
PIAS1
CLCF1
SOCS5
SOCS2
GHR
PKD2
STAT5A
STAT1
SOCS3
CCL2
PKD1
JAK2
SOCS1
CCR2
FGFR3
NMI
STAT2
IL31RA
PIAS4
STAT5B
PRLR
STAT4
GH
SH2B2
IFNAR2
AGER
STAT3
1.5
1.0
0.5
0.0
-0.5
-1.0
-1.5
SMAD cascade
T
GATA4
NODAL
BMP7
BMP6
HNF4A
CITED1
HIPK2
TRF
AFP
BMP4
JUN
SKI
BMP2
FOS
B
irr d3
irr d7
non-irr.
DMSO
JAKi I
Noggin
DMSO
JAKi I
Noggin
GFAP
α-tubulin
STAT3-pY705
STAT3
SMAD1-pS465
SMAD1
Vinculin
C
Cyclin B1
Cyclin A2
Ki67
Gene expression rel. to NSC non-irr
10
1
0.1
0.01
irr d7
irr+recNoggin d7
Ki67
CycB1
Tuj1
irr d7
irr+JAKi d7
D
% SA-β-gal+ cells
100
80
60
40
20
0
non-irr
irr d7
irr+JAKi d7
irr+recNoggin d7
E
non-irr
irr d7
JAKi irr d7
DNA
GFAP
γH2AX
F
non-irr
irr 1h
irr d7
irr d7, re-irr 1h
JAKi
-
+
← pChk2
← Chk2
p53-pS15
p53
STAT3-pY705
Vinculin

Figure S4

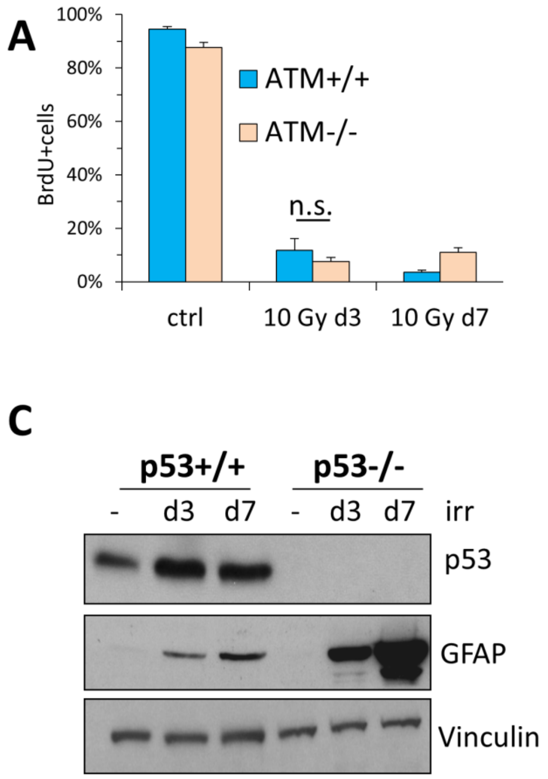
A
100%
80%
60%
40%
20%
0%
BrdU+cells
ATM+/+
ATM-/-
n.s.
ctrl
10 Gy d3
10 Gy d7
B
100%
80%
60%
40%
20%
0%
BrdU+cells
p53+/+
p53-/-
n.s.
ctrl
10 Gy d3
10 Gy d7
C
p53+/+
p53-/-
- d3 d7 - d3 d7 irr
p53
GFAP
Vinculin

Figure S5

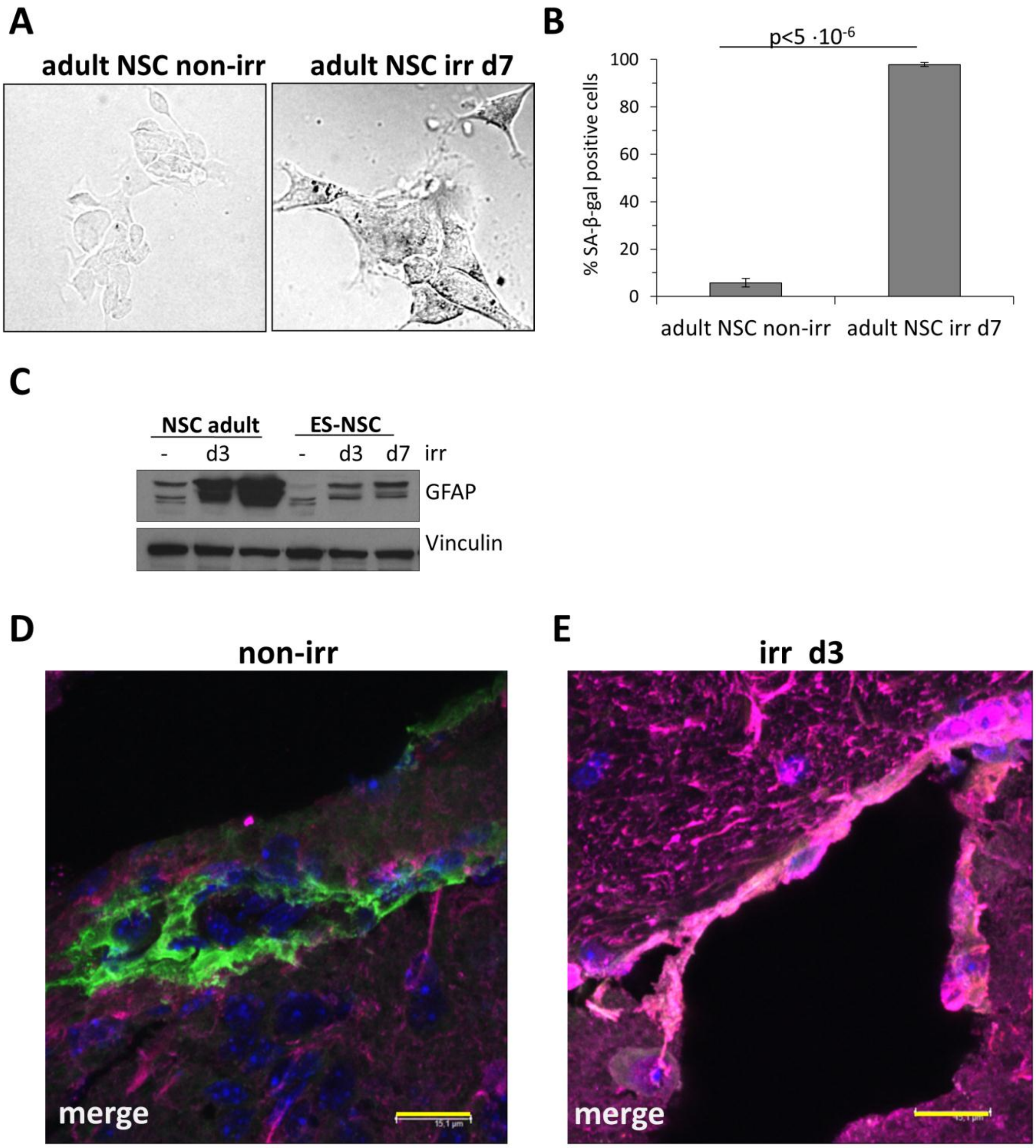
A
adult NSC non-irr
adult NSC irr d7
B
p<5 ·10-6
% SA-β-gal positive cells
0
20
40
60
80
100
adult NSC non-irr
adult NSC irr d7
C
NSC adult
ES-NSC
-
d3
-
d3
d7
irr
GFAP
Vinculin
D
non-irr
merge
E
irr d3
merge

Figure S6

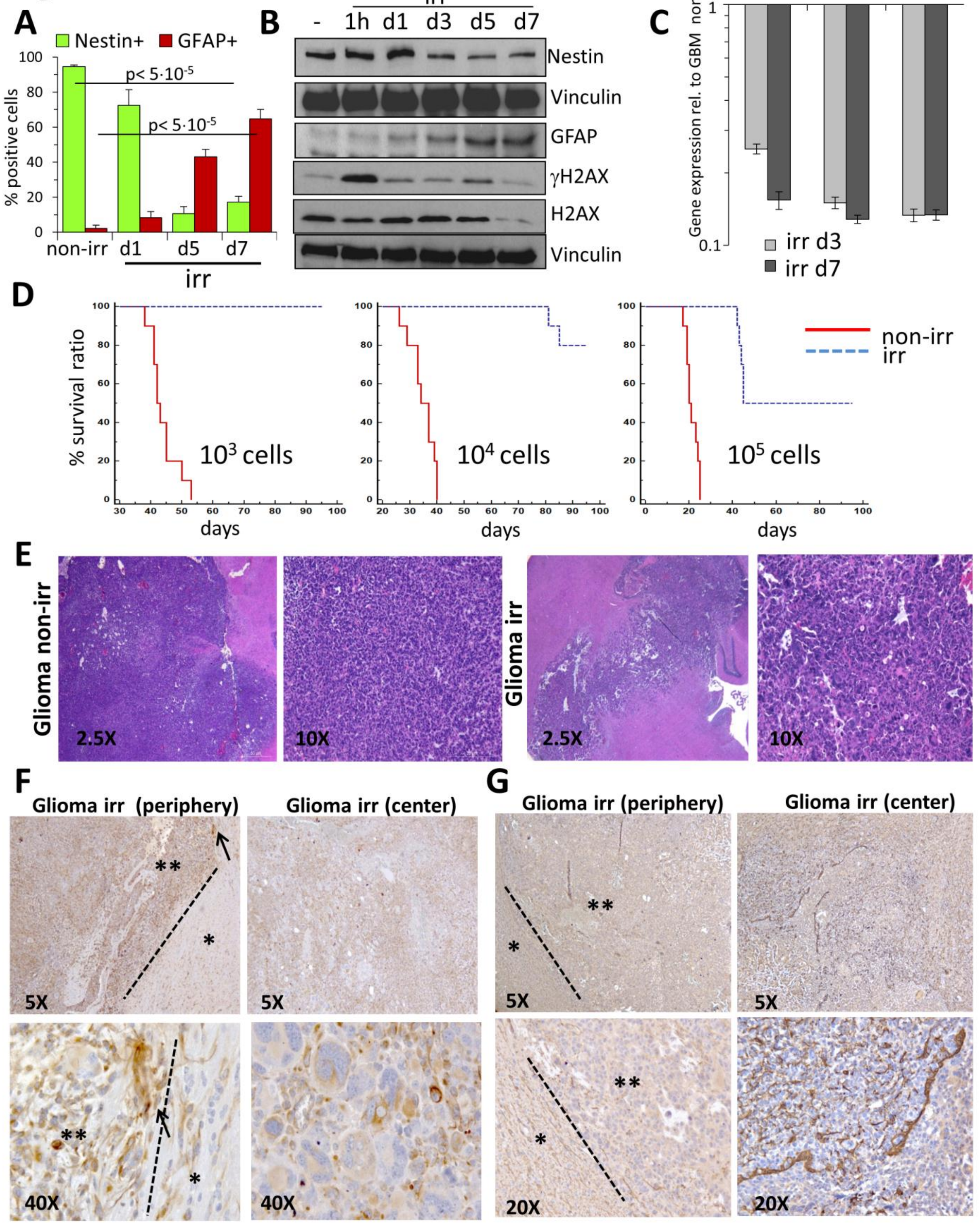
A
Nestin+
GFAP+
% positive cells
p< 5·10-5
p< 5·10-5
non-irr
d1
d5
d7
irr
B
irr
-
1h
d1
d3
d5
d7
Nestin
Vinculin
GFAP
γH2AX
H2AX
Vinculin
C
Cyclin B1
Cyclin A2
Ki67
Gene expression rel. to GBM non-irr
irr d3
irr d7
D
% survival ratio
10^3 cells
10^4 cells
10^5 cells
days
non-irr
irr
E
Glioma non-irr
Glioma irr
2.5X
10X
F
Glioma irr (periphery)
Glioma irr (center)
5X
40X
G
Glioma irr (periphery)
Glioma irr (center)
5X
20X

Supplemental Figure Legends

Fig. S 1 X-ray irradiation of NSC leads to a senescence-like cell cycle arrest, yet associated with DNA repair and progressive downregulation of DDR signaling

(A) A simplified scheme of DNA damage response signaling and the role of ATM. Appearance of DNA double strand breaks (DSBs) leads to autophosphorylation and activation of the apical Serine/Threonine (S/T) kinase ATM. ATM then phosphorylates at their pS/TQ sites several protein targets, which can be described either as 1) DSB-associated factors, which can be detected as nuclear foci labeling DSBs (e.g., 53BP1 and the S139-phosphorylated Histone H2A isoform γH2AX), or as 2) diffusible factors which can either transcriptionally regulate other genes (e.g., p53) or target additional proteins (e.g. the kinase CHK2). Ultimately, the activation of DDR results in DNA repair, or in case Western blot analysis characterizing the proliferation arrest in irradiated NSC. Cell cycle dependent kinase (CDK) inhibitors $p21^{CIP}$ and $p27^{KIP}$ were probed; furthermore Rb protein became hypo-phosphorylated in the time course after irradiation. Loading was normalized for α-tubulin.

(B) Representative transmission microscopy images of irradiated NSC displaying senescence-associated-β-galactosidase activity, magnification: 20x.

(C) One hour after irradiation, NSC uniformly displayed the assembly of autophosphorylated ATM (S1981) and 53BP1 foci, as analyzed by confocal microscopy of immunofluorescence stainings. Note that the majority of cells lost the signal as shown for day 7 after irradiation. Bar: 15µm. Quantifications of DDR positive cells are provided on the right-hand side.

(D) DSB detection by neutral COMET assay in control NSC and cells 1h and 7 days after irradiation. Olive tail moments are presented as box plot diagram, with vertical bars

indicating median values. Error bars show standard deviation (SD). *p*-values of the median values was calculated using Dunn's method.

Fig. S 2 Irradiation-induced astrocytic differentiation phenotype becomes more robust over time.

(A) Quantification of a time course study of GFAP expression in irradiated NSC as detected by immunostaining and FACS. Error bars show SD.

(B) Immunofluorescence analysis of SOX2 expression in non-irr NSC and cells on day 7 post irr. DNA was stained with DAPI. Representative wide field microscopy images are shown, magnification: 40x.

(C) Immunofluorescence analysis of the nuclear SOX9 signal in non-irr NSC and cells on day 7 post irr. DNA was stained with DAPI. Representative wide field microscopy images are shown, magnification: 40x.

(D) Immunofluorescence analysis of the cell membrane protein Aquaporin 4 (AQP4) expression in non-irr NSC and cells on day 7 post irr. DNA was stained with DAPI. Representative wide field microscopy images are shown, magnification: 40x.

(E) Western blot analysis of irr NSC for the expression of OLIG2 and GFAP. Loading was normalized for vinculin.

(F) Quantitative TaqMan based RT-PCR analysis of NSC on day 14 post irradiation for the expression of typical markers of NSC (*Nestin*, *PAX6*), astrocytes (*GFAP, S100β*), neuronal precursors (*DCX)*, mature neurons (*TUJ1*) and oligodendrocyte precursors (*PDGFRα*), normalized against non-irr NSC. *18S* rRNA was used as housekeeping gene, error bars show SD.

(G) qRT-PCR analysis of NSC 7 days and 1 month after irradiation, normalized against non-irr NSC. Note that cells upregulated the astrocytic marker *S100β* at the later time

point, while *LIF* induction did not persist (unlike *BMP2/4*). *B2M* was used as housekeeping gene, error bars show SD.

(H) NSC were subjected 8h after irradiation (irr) to neuronal differentiation protocol (Spiliotopoulos et al., 2009). In parallel, non-irradiated NSC were similarly treated to neural differentiation. Neurons were scored at the end of the process for the typical marker TUJ1 (also known as β-Tubulin III). Note the equal neuronal differentiation proficiency of irr and non-irr NSC under neurogenic conditions.

Fig. S 3 Inhibition of BMP2/JAK-STAT signaling in irradiated NSC suppresses differentiation and reveals residual DDR activity, but does not allow proliferative recovery from senescence.

(A) Microarray analysis and heat-map of JAK/STAT and SMAD cascade responsive genes obtained from gene sets from Gene Ontology classes (Ashburner et al., 2000). The distance between two gene expression profiles is 1 when the two profiles are identical, and decreases towards 0 as the difference between them increases.

(B) Western blot analysis on the effects of JAK/STAT signaling inhibition (JAKi) and the inhibitor of BMP2/4 receptor binding (Noggin) on irradiation induced differentiation. Note the loss of GFAP induction in irradiated NSC when continuously treated with JAKi or Noggin. Loading was normalized for α-tubulin and vinculin.

(C) qRT-PCR analysis of cell cycle arrest of NSC on day 7 post irradiation, with or without continuous Noggin (left-hand panel) or JAKi (right-hand panel) treatment. *TUJ1* (*β-tubulin III*) expression was tested in JAKi-treated cells to address a possible neural differentiation. *B2M* was used as housekeeping gene, error bars show SD.

(D) Assay for senescence-associated β-galactosidase activity on NSC on day 7 post irradiation, with or without continuous JAKi or Noggin treatment. Positive cells showing the characteristic blue signal were scored by transmission microscopy.

(E) Representative confocal images of control NSC and cells on day 7 post irradiation, with or without continuous JAKi treatment. Residual DDR activity was detected by immunofluorescence analysis for γH2AX foci retention. DNA was stained with DAPI. Bar: 20µm

(F) Western blot analysis of residual DDR activity in irradiated NSC, treated with JAKi. Last two lanes show irradiated cells at d7, re-irradiated (re-irr) and analysed after 1h. Note the persistent phosphorylation of Chk2 and increased total p53 levels in irradiated cells when continuously treated with JAKi. Loading was normalized for vinculin.

Fig. S 4 Cell cycle arrest and apoptosis in p53 and ATM deficient NSC and the impact of pharmacological inhibition of ATM and p53.

(A) BrdU incorporation based assay for DNA replicating activity of irradiated ATM deficient and isogenic wild type NSC. BrdU was supplied at 3.3 µM in the culture medium 24h prior to fixation and detected by immunofluorescence and wide-field microscopy. n.s.; not significant. Error bars show SD.

(B) BrdU incorporation based assay for DNA replicating activity of irradiated p53 deficient and isogenic wild type NSC. BrdU was supplied at 3.3 µM in the culture medium 24h prior to fixation and detected by immunofluorescence and wide-field microscopy. n.s.; not significant. Note that p53-/- cells still efficiently arrested after irradiation, however, a slight increase in proliferation on day 7 reflected the clonal colonies of escapees. Error bars show SD.

(C) Western blot analysis of GFAP induction in irradiated wild type and isogenic p53-deficient NSC. Membrane was also probed for p53 protein presence and loading normalized for vinculin.

Fig. S 5 Adult NSC enter cellular senescence upon irradiation and strongly upregulate GFAP.

(A) Representative transmission microscopy images of non-irr adult NSC and cells at day 7 post irr displaying senescence-associated-β-galactosidase activity in the latter, magnification: 20x.

(B) Quantification of adult NSC positive for senescence-associated-β-galactosidase activity before and at day 7 post irr.

(C) Western blot analysis of murine adult forebrain NSC and ES-derived NSC for GFAP expression upon irradiation. Loading was normalized for vinculin.

(D) Merged collapsed Z-Stack of all four channels as shown separately in Fig 7D. Bar: 15µm

(E) Merged collapsed Z-Stack of all four channels as shown separately in Fig 7E. Bar: 15µm

Fig. S 6 Mouse glioblastoma stem cells lose Nestin expression and undergo astrocytic differentiation upon irradiation *in vitro* and *in vivo*.

(A) Quantification of a time course study of Nestin expression in irradiated GL261 cells, grown in adherent conditions, as detected by immunofluorescence and wide-field microscopy. Error bars show SD.

(B) Western blot analysis of adherently grown GL261-CSC for the Nestin and GFAP expression and γH2AX signal upon irradiation. Loading was normalized for vinculin.

(C) qRT-PCR analysis of GL261 cells on day 3 and day 7 after irradiation for the expression of proliferation markers *Cyclin B1, Cyclin A2* and *Ki67. B2M* was used as housekeeping gene, error bars show SD.

(D) Non-irr (red line) or irr (dashed blue line) GL261-CSC at 3 increasing cell quantities were injected in xenograft assays into the nucleus caudatum of mice to form GBM

tumors. Kaplan-Meier curves showing the survival of host animals is shown. Note the reduced mortality (down to zero) when irr cells were injected.

(E) Hematoxylin and eosin (H&E) staining of non-irr GBM xenograft (left panels) and GBM after radiation-therapy (right panels). Note that despite a shrunken tumor mass a prominent portion of the xenograft has survived irradiation. Magnification: top panels: 2.5x, bottom panels: 10x.

(F) Representative immunohistochemistry analysis of Nestin expression in irradiated GBM tumors at d30 (=d20 after irr at d10). Note that a prominent Nestin signal indicating self-renewing cells (arrow) could be observed in tumor periphery (left panels; * healthy tissue, ** tumor, separated by a dashed line), but not at tumor center (right panels). Magnification: top panels: 5x, bottom panels: 40x.

(G) Representative immunohistochemistry analysis of GFAP expression in irradiated GBM tumors at d30 (=d20 after irr at d10). Note that in advanced tumors the differentiation marker GFAP could be observed only in the central glioma mass (right-hand panels), but not in the expanding tumor periphery, which hardly showed any GFAP signal (left-hand panels; * healthy tissue, ** tumor, separated by a dashed line). Magnification: top panels: 5x, bottom panels: 20x.

Supplemental Tables

Table S1

Effects of candidate JAK-STAT signaling cytokines on the astrocytic differentiation of NSC

Candidate JAK-STAT activating /GFAP inducing cytokine	Reference	Experimental observations
**LIF (leukemia inhibitory factor)**	(Taga and Fukuda, 2005; Turnley and Bartlett, 2000)	Expression lost in irr NSC at later time points, not induced at all in irr p53-/- NSC.
**CNTF (ciliary neurotrophic factor)**	(Turnley and Bartlett, 2000)	Not induced in irr p53-/- NSC
**CTF-1 (cardiotrophin 1)**	(Turnley and Bartlett, 2000)	Not induced in irr p53-/- NSC
**OsM (oncostatin M)**	(Turnley and Bartlett, 2000)	Remains undetectable even after irr
**IL-6 (interleukin 6)**	(Taga and Fukuda, 2005)	Late induction after irr (GFAP already upregulated), almost no effect on GFAP induction

Table S2

Survival ratios of mice after cranial injection of non-irradiated GL261-GCS

**GL261 non-irr**	Number of injected cells		
	**10^5**	**10^4**	**10^3**
Survival Mean, days ±SD	21.3±2.8	34.8±4.7	44.0±4.5
Survival Median, days	20.5	35.5	42.5

Table S3

Tumor incidence and mortality in mice after cranial injection of irradiated GL261-GCS

**Tumor incidence and Mortality**	Number of injected cells		
	**10^5**	**10^4**	**10^3**
GL261 non-irr	10/10	10/10	10/10
GL261 irr	5/10	2/10	0/10

Table S4

Statistics checklist for main and supplemental data

Figure Panel	Test	S.D or S.E.M	n value	independent experiments /technical replicates	*p* values provided?
**1a**	t-test, 2-tailed	s.d.	3	*independent experiments*	yes, for the first significant change in kinetics
**1c**	t-test, 2-tailed	s.d.	3	*independent experiments*	yes, for the first significant decrease in kinetics
**1f**	none	s.d.	3	*qRT-PCR technical triplicates*	not applicable
**2a**	t-test, 2-tailed	s.d.	3	*independent experiments*	yes
**2c**	t-test, 2-tailed	s.e.m.	4	*independent experiments*	yes, ctrl vs d3 and d7
**2d**	t-test, 2-tailed	s.d.	3	*independent experiments*	yes
**2f**	t-test, 2-tailed	s.e.m.	≥6	*independent experiments*	yes
**2g**	none	s.d.	3	*qRT-PCR technical triplicates*	not applicable
**2i**	none	s.d.	3	*qRT-PCR technical triplicates*	not applicable
**3a**	none	s.d.	3	*qRT-PCR technical triplicates*	not applicable
**3c**	none	s.d.	3	*qRT-PCR technical triplicates*	not applicable
**3e**	none	s.d.	3	*independent experiments*	no sign. increase in BrdU+ after inhibitor treatment
**4b**	none	s.d.	3	*qRT-PCR technical triplicates*	not applicable
**4d**	none	s.d.	1	*representative experiment*	not applicable
**4e**	none	s.d.	3	*qRT-PCR technical triplicates*	not applicable
**4f**	none	s.d.	3	*qRT-PCR technical triplicates*	not applicable
**5b**	none	s.d.	3	*qRT-PCR technical triplicates*	not applicable
**5d**	none	s.e.m.	≥ 2	*independent experiments*	not applicable
**6b**	t-test, 2-tailed	s.d.	3	*independent experiments*	yes
**6c**	none	s.d.	3	*qRT-PCR technical triplicates*	not applicable
**6f**	Mann-Whitney Rank Sum Test	s.e.m.	3	*independent experiments (details see legend)*	yes
**7b**	none	s.d.	3	*qRT-PCR technical triplicates*	not applicable
**7c**	none	s.d.	1	*serial dilution experiment*	not applicable
**7d**	t-test, 2-tailed		3	*Kaplan-Meyer mortality curve*	yes
**7f**	t-test, 2-tailed	s.d.	3	*triplicates of 5 section fields from 2 independent animals each*	yes
**S1d**	t-test, 2-tailed	s.d.	3	*independent experiments*	yes, for the first significant decrease in kinetics
**S1e**	Dunn's method	s.d.	3	*independent experiments*	yes
**S2a**	t-test, 2-tailed	s.d.	3	*independent experiments*	yes, ctrl vs d7
**S2f**	none	s.d.	3	*qRT-PCR technical triplicates*	not applicable
**S2g**	none	s.d.	3	*qRT-PCR technical triplicates*	not applicable
**S2h**	t-test, 2-tailed	s.d.	3	*independent experiments*	yes, p value not significant
**S3c**	none	s.d.	3	*qRT-PCR technical triplicates*	not applicable
**S3d**	none	s.d.	1	*representative experiment*	not applicable
**S4a**	t-test, 2-tailed	s.d.	3	*independent experiments*	p values on d3 not significant between wt and KOs
**S4b**	t-test, 2-tailed	s.d.	3	*independent experiments*	p values on d3 not significant between wt and KOs
**S5b**	t-test, 2-tailed	s.d.	3	*independent experiments*	yes
**S6a**	t-test, 2-tailed	s.d.	3	*independent experiments*	yes
**S6c**	none	s.d.	3	*qRT-PCR technical triplicates*	not applicable

Supplemental Experimental Procedures

Derivation of NSC

Murine embryonic stem cells (ESC) were grown in feeder-free conditions on high-glucose DMEM, supplemented with 15% fetal calf serum (FCS, PAN Biotech), recombinant LIF supernatant, 0.1 mM β-mercaptoethanol, 2mM L-glutamine (L-Gln), 100U/ml penicillin and 100μg/ml streptomycin (P/S). In the first step, ESC were seeded on gelatinized dishes in N2B27 culture medium (1:1 DMEM/F12 and Neurobasal medium (Invitrogen), supplemented with 0.5x N2 supplement (Invitrogen) and 0.5x B27 supplement (Invitrogen), 0.1 mM β-mercaptoethanol, L-Gln and P/S. Medium was changed daily without passaging for 7 days. In the second step, colonies were gently dislodged using Accutase (Sigma Aldrich), washed with PBS, gently resuspended in NSC culture medium (see below), then transferred at high density into culture flasks. There, after 2 days of culturing, formation of neurospheres could be observed. Neurospheres were disrupted by Accutase treatment and gentle pipetting and transferred to cell culture dishes in NSC culture medium. Adherently outgrowing NSC were expanded into stable cell lines through several passages (≥20) before being used for experiments.

Following gene-deficient ESC lines were used together with isogenic wild types to derive gene-deficient NSC: BMP2-/- (Castranio and Mishina, 2009), kindly provided by Trisha Castranio and Yuji Mishina (NIEHS-NIH, USA and U. of Michigan, USA, respectively); ATM-/- (Xu and Baltimore, 1996), kindly provided by Yang Xu (UCSD, USA); p53-/- (Sabapathy et al., 1997), kindly provided by Jean-Christophe Marine (VIB, Belgium).

Cell culture of NSC

Murine ES-derived NSC as well as adult mouse forebrain NSC (Conti et al., 2005), kindly provided by Luciano Conti, were grown in Euromed-N cell culture medium (Euroclone), supplemented with L-Gln and P/S, 1x N2 supplement (Invitrogen), 20ng/ml each murine EGF and FGF2 (ProSpec, Israel) at 5% CO_2 and 37° C. For adult forebrain NSC, cell culture dishes were coated with 0.2% porcine gelatin (Sigma Aldrich), glas slides were coated with 5mg/ml laminin (Santa Cruz) and 0.1% gelatin.

Cell culture of GBM cells

Murine glioblastoma cell line Gl261-CSC was grown either in DMEM/F12 medium supplemented with L-Gln and P/S and either 20% FCS or, where referred to serum-free culture, with 0.5x B27 supplement (Invitrogen) and 20ng/ml each human EGF and FGF2 (Peprotech). For adherent serum-free culture dishes were covered with 10 ug/ml laminin.

***In vivo* limiting dilution**

GL261 were seeded overnight onto 6-well plates at $2x10^5$/well and irradiated with 10 Gy dose using a Faxitron RX-650 device. Three days after irradiation equal cell number from non-irr and irr cells were injected into C57BL6N mice at three different doses (10^5, 10^4, or 10^3; n=10 mice/dose/group, total 60 mice).The stereotactic coordinates with respect to the bregma are: 0.7 mm posterior, 3 mm left lateral, 3.5 mm deep into the nucleus caudatum. Cumulative survival curves were constructed by Kaplan–Meier method using MedCalc 9.3 software. Statistical comparisons of data sets were performed by a two-tailed Student’s T-test and results were considered significant at $p < 0.05$.

Animal treatment

Mice were monitored every day until sacrifice, in accordance with current directives of the Institution and Minister of Health (Italy). All irradiations as well sacrificing/perfusions were performed on anaesthetized animals using 2,2,2-Tribromoethanol solution (Avertin, Sigma Aldrich, 0.1ml/10g body weight).

For *in vivo* cell fate tracing, adult male mice of SOX2-CreERT2 / R26::loxP-stop-loxP::YFP background and 6 months of age were treated with 0.1 mg/g body weight of Tamoxifen (#T5648, Sigma Aldrich) for 6 days intraperitoneally, one injection/day (20 mg/ml in Ethanol/Corn Oil 1:10). Exclusive cranial irradiation was achieved by the use of a lead body protection cover, irr of 10Gy was performed in the Gilardoni RADGIL irradiator (30.3min). For perfusion, anesthetized mice were transcardially perfused with 30 ml of 0.1% heparin/PBS followed by 4% PFA/PBS (Sigma Aldrich). Whole brains were post-fixed in 4% PFA/PBS overnight, cryoprotected in 10% sucrose (w/v in in PBS) overnight, in 20% sucrose overday and in 30% sucrose overnight, then snap frozen in cold isopentane in liquid nitrogen. Brain tissues were imbedded into Killik compound (#059801, Bio-Optica) and cut on a Leica CM1900 cryostat into 10μm serial sections and used for Hematoxylin/Eosin (H&E) staining or immunofluorescence analysis.

Immunofluorescence microscopy

Cells grown on glass cover slips were fixed in methanol-acetone (1:1) for DDR studies, otherwise in 4% paraformaldehyde (PFA) and permeabilized with 0.2% Triton X100. After blocking with 0.5% BSA and 0.2% gelatin in PBS, cells were probed with appropriate primary antibodies and Alexa-fluor 488- and 647-labeled secondary antibodies (Invitrogen). Nuclear DNA was stained by DAPI (Sigma Aldrich). For BrdU detection, incubation with

DNAse I (NEB) was performed together with the primary anti-BrdU antibody (#347580, BD Biosciences) in order to retrieve the epitope.

Following primary antibodies were used for immunofluorescence studies: Nestin (#MAB353, Millipore), GFAP (#Z0334 Dako and # ab4674 Abcam), (pS/TQ (phospho-(Ser/Thr) ATM/ATR Substrate Antibody #2851, Cell Signaling), 53BP1 (#NB100-304, Novus Biologicals), γH2AX (#05-636, Millipore), SOX2 (#09-0024, Stemgent), SOX9 (#sc-17341, Santa Cruz), Aquaporin 4 (#ABIN671181, antibodies-online), s100β (#S2644, Sigma Aldrich), GFP/YFP (#AM11009PU, Acris Antibodies), BrdU (#347580, BD Biosciences), ATM-pS1981 (#200-301-400, Rockland), 53BP1 (#NB100-304, Novus Biologicals), TUJ1 (#MRB-435P, Covance). Confocal images were obtained with a Leica TCS SP2 AOBS confocal laser microscope by sequential scanning and processed with Leica LAS AF Lite software; wide field images with an Olympus AX70 upright microscope and processed with ImageJ software.

The colocalisation ratio of the YFP signal in the brain tissue with astrocyte markers GFAP and S100β was calculated for each layer of the Z-stack, using ImageJ and the JACoP plug-in (Bolte and Cordelieres 2006).

SA-β-galactosidase assay

Cells were fixed for 3 min in 4% PFA, washed with PBS and immediately incubated overnight at 37°C in the pH 6.0 assay buffer (440mM NaH_2PO_4, 60mM Na_2HPO_4, 150mM NaCl, 2mM $MgCl_2$) containing 40mM $K_3[Fe(CN)_6]$, 40mM $K_4[Fe(CN)_6]$ and 1mg/ml 5-bromo-4-chloro-indolyl-β-D-galactopyranoside (X-Gal). Images were acquired with Olympus BX51 upright microscope.

Immunohistochemistry

Paraffin-embedded tumor sections were prepared according to standard procedures, blocked with 5% goat serum in PBS for 60 min, incubated overnight with primary antibodies (Nestin, # MAB2736, R&D Systems and GFAP, #Z0334, DAKO) and biotinylated secondary antibodies (Vector Lab). Antibody binding was detected using the Vectastain Elite Avidin–Biotin Complex-Peroxidase kit according to the manufacturer's instructions. All sections were counterstained with Mayer's hematoxylin and acquired using Leica MDLB light microscope.

Immunoblotting

Cells were lysed in NP40 lysis buffer (1% NP40, 50mM Tris-Cl pH 8, 150mM NaCl, 2mM EDTA, 1mM DTT, 1mM NaF, 100µM Na_2VO_4 and protease inhibitor cocktail (Roche) and 40µg of whole cell lysate in Lämmli loading buffer were resolved by SDS-PAGE, transferred to nitrocellulose membranes (Protran) using Biorad electrophoresis systems and probed with primary and secondary antibodies in 5% bovine serum albumin (BSA) and skimmed milk, respectively. Following primary antibodies were used: Nestin (#611658, BD Biosciences), GFAP (#Z0334 Dako), PAX6 (#PRB-278P, Covance), ATM-pS1981 (#200-301-400, Rockland), ATM (#A1106, Sigma Aldrich), p53-pS15 (#9284, Cell Signaling), p53 (#K0181-3, MBL), γH2AX (#05-636, Millipore), H2AX (#ab20669, Abcam), CHK2 (#05-649, Millipore), STAT3-pY705 (#9138, Cell Signaling), STAT3 (#AP02587PU, Acris), JAK2-pY1007/1008 (#sc-16566, Santa Cruz), SMAD1-pS465 (#AP08024PU, Acris), α-tubulin (#T6199, Sigma Aldrich), Vinculin (#V4505, Sigma Aldrich), $p27^{KIP}$ (Santa Cruz, #sc-1641,), Rb (BD, #554136), OLIG2 (#ab110031, Abcam). HRP-coupled secondary antibodies (Sigma Aldrich) and ECL Plus™ Western Blotting Detection Reagents and X-ray films (Amersham) were used for signal detection.

Gene expression analysis

Total RNA was extracted from live cells with Trizol reagent (Invitrogen), precipitated with isopropanol and ethanol and dissolved in DEPC-treated water (Invitrogen). 1 µg of total RNA (as quantified with NanoVue device, General Electric (GE)) was used for retrotranscription using VILO reverse transcription kit (Invitrogen) according to manufacturer's instructions and without RNAse treatment. RT-minus reactions (without reverse transcriptase enzyme) were also prepared. Estimated 20ng of cDNA in 25µl reaction volume were analyzed in triplicate by quantitative RT-PCR amplification on a Light Cycler 480 system (Roche) using SYBR Green assay (QuantiFast SYBR Green PCR Kit, Qiagen) according to manufacturer's instructions and for 40 cycles. CT-values were obtained by calculation of the second derivative using Light Cycler 480 software (Roche) and normalized among samples against a housekeeping gene β-2-microglobulin (B2M). RT-minus preparations proved to be negative. Following forward and reverse primers (FP and RP) were designed with Roche UniversalProbe Library online software against *Mus musculus* and used in supplementary experiments:

B2M: FP: CTGCAGAGTTAAGCATGCCAGTA; RP: TCACATGTCTCGATCCCAGTAGA
NESTIN: FP: CTGCAGGCCACTGAAAAGTT; RP: TCTGACTCTGTAGACCCTGCTTC
SOX2: FP: TGCTGCCTCTTTAAGACTAGGG; RP: TCGGGCTCCAAACTTCTCT
PAX6: FP: GTTCCCTGTCCTGTGGACTC; RP: ACCGCCCTTGGTTAAAGTCT
GFAP: FP: TGGAGGAGGAGATCCAGTTC; RP: AGCTGCTCCCGGAGTTCT
BMP2: FP: CGGACTGCGGTCTCCTAA; RP: GGGGAAGCAGCAACACTAGA
BMP2 (Fig. 4E,F): FP: AGATCTGTACCGCAGGCACT; RP: GTTCCTCCACGGCTTCTTC
BMP4: FP: GAGGAGTTTCCATCACGAAGA; RP: GCTCTGCCGAGGAGATCA
LIF: FP: AAACGGCCTGCATCTAAGG; RP: AGCAGCAGTAAGGGCACAAT
OLIG2: FP: AGACCGAGCCAACACCAG; RP: AAGCTCTCGAATGATCCTTCTTT
SOX9: FP: GTACCCGCATCTGCACAAC; RP: CTCCTCCACGAAGGGTCTCT
MSI1: FP: GATGCCTTCATGCTGGGTAT; RP: CGTAGGTCGTGGCTTGGA
VIMENTIN: FP: CCAACCTTTTCTTCCCTGAA; RP: TGAGTGGGTGTCAACCAGAG
ARS2: FP: GGCAGCAGATGCAGGACT; RP: GGGTGGTACTTAGATCGGAACC
ATM: FP: TGCAGATTTATATCCATCATCCAC; RP: TTTCATGGATTCATAAGCACCTT

53BP1: FP: AAAGTCTGCCACCGTGAAAC; RP: TCTCCAGTCTCACAGGGACTC
MRE11: FP: CTTTTTCAGGCACAGGGAAC; RP: TGTGATGAGCATCCCAAAGT
MDC1: FP: AGGGCAGCTACGTCTCTTCA; RP: CCAAGGTAGAGGGGGAAATC
CHK2: FP: TTATTCCTGAAGTCTGGACAGATG; RP: CTAACAGTTTCTTGACAAGGTCCA
p53: FP: ACGCTTCTCCGAAGACTGG; RP: AGGGAGCTCGAGGCTGATA
DNA-PKcs: FP: TGCAGAGAAATGTGATTGCAC; RP: CCACGGTGGAAGATCTTTTG
ATR: FP: CATCCAGAATTAGAAAAGATAAAAGCA; RP:
GAAGATCAGTAGTCTCAGAGGTTTCC
PARP1: FP: AGGCCGCCTACTCTATCCTC; RP: GATTCACTGCTGCCTTGAGA
H2AX: FP: AAGCCGGTGAATCCCTGT; RP: AGCTGCAAAAGTTCCAGTTCA
S100β: FP: AACAACGAGCTCTCTCACTTCC; RP: CTCCATCACTTTGTCCACCA
Ki67: FP: GCTGTCCTCAAGACAATCATCA; RP: GGCGTTATCCCAGGAGACT
CYCLIN B1: FP: GCGCTGAAAATTCTTGACAAC; RP: TTCTTAGCCAGGTGCTGCAT
CYCLIN A2: CTTGGCTGCACCAACAGTAA; RP: CAAACTCAGTTCTCCCAAAAACA
TUJ1: GCGCATCAGCGTATACTACAA; RP: CATGGTTCCAGGTTCCAAGT

The facility-based TaqMan assay (Fig. S 2F) was performed on ABI PRISM® 7900HT Sequence Detection System using TaqMan primer pairs (Applied Biosystems) for *B2M* (Mm00437762_m1), *Nestin* (Mm00450205_m1), *GFAP* (Mm01253033_m1), *PAX6* (Mm00443072_m1), *DCX* (Mm00438401_m1), *S100β* (Mm00485897_m1), *TUJ1* (*tubb3*, Mm00727586_s1), *PDGFRα* (Mm00440701_m1).

Flow cytometry (Fluorescence-activated cell sorting, FACS)

For GFAP detection, cells were fixed in 75% ethanol (1h, 4°C),) washed with 1% BSA in PBS and stained with rabbit-anti-GFAP antibody (#Z0334 Dako), followed by Alexa-fluor-488 labeled secondary antibody (Invitrogen). Cells stained with secondary antibodies only were used as negative controls. FACS acquisition and analysis were performed on BD FACScalibur using CellQuest software.

Microarray analysis

Hybridized arrays were stained and washed (GeneChip Fluidics Station 450) and scanned (GeneChip Scanner 3000 7G). Cell intensity values and probe detection calls were computed using the Affymetrix GeneChip Operating Software (GCOS). Further data processing was performed in the R computing environment (http://www.r-project.org/) version 2.8.0 with BioConductor packages (http://www.bioconductor.org/). Robust Multi-Array Average (RMA) normalization was applied (Irizarry et al., 2003). Data were then filtered based on Affymetrix detection call and probeset intensity, so that only probesets that had a present call and intensity value >100 in at least one of the arrays were retained. Statistical analysis was performed with Limma software (Smyth, 2004). P-values were adjusted for multiple testing using Benjamini and Hochberg's method to control the false discovery rate (Hochberg and Benjamini, 1990). Genes with adjusted P values below 0.05 were considered differentially expressed. A threshold value of 2-fold change was also applied. Functional clustering of the differentially expressed genes was carried out through the Database for Annotation, Visualization, and Integrated Discovery (DAVID; (Huang da et al., 2009)).

For each gene of interest, the expression values were normalized (Z-transform) to calculate the average and standard deviation across conditions and by subtracting the average value and dividing by the standard deviation, leading for each gene to a new set of expression values with average 0 and standard deviation 1. Similarities were calculated between all pairs of gene expression using a formula:

$$Similarity(G_i, G_j) = \frac{1}{n} * \sum_{q=1}^{n} \frac{1}{1 + \left|G_i^q - G_j^q\right|}$$

The similarities between two profiles were multiplied by their Pearson correlation coefficient, and transformed into a distance measures by subtracting them from 1, which were used to generate a dendrogram (based on the complete linkage algorithm). A matrix was generated (heatmap) in which columns and conditions (rows) with similar gene expression profiles were close to each other.

Some of the categories analyzed were obtained from Gene Ontology: the JAK-STAT cascade, the SMAD protein signal transduction, and the DDR signaling category. The latter was identified by pooling together different Gene Ontology categories (DNA Replication, Recombination and Repair, p53 signaling, ATM signaling) and further integrated with genes found in literature (Jackson and Bartek, 2009). The category of Astrocytes was built entirely from literature (Cahoy et al., 2008; Obayashi et al., 2009).

COMET assay

Assay was performed with CometSlide kit (Trevigen) according to manufacturer's instructions for neutral electrophoresis. Between 100 and 200 cells were scored in triplicate for each experimental condition. COMET tails were analyzed with CometScore 1.5 software, statistical significance of Olive tail moment medians was calculated using Dunn's Method for multiple comparisons versus control group.

Neuron differentiation

A modified neuron differentiation protocol from (Spiliotopoulos et al., 2009) was adjusted to the conditions of irradiation. NSC were seeded 12h prior to irradiation on laminin (3mg/ml, Santa Cruz)-coated glass cover slips in standard NSC culture medium. 8h after 10Gy irradiation (or mock-irradiation) medium was replaced with medium A (1:3 DMEM/F12 and Neurobasal), supplemented with 1.5x B27 and 0.5x N2 and10 ng/ml FGF2 and 20ng/ml

BDNF (ProSpec, Israel), L-Gln and P/S). After 3 days, medium A was switched to medium B (similar to A, but with 6.7 ng/ml FGF2 and 30ng/ml BDNF). Cells were cultured for 5 days in medium B prior to fixation.

Supplemental References

Ashburner, M., Ball, C.A., Blake, J.A., Botstein, D., Butler, H., Cherry, J.M., Davis, A.P., Dolinski, K., Dwight, S.S., Eppig, J.T., *et al.* (2000). Gene Ontology: tool for the unification of biology. Nature genetics *25*, 25-29.

Bolte, S., and Cordelieres , F.P. (2006). A guided tour into subcellular colocalization analysis in light microscopy. Journal of Microscopy *224*, 213-232.

Cahoy, J.D., Emery, B., Kaushal, A., Foo, L.C., Zamanian, J.L., Christopherson, K.S., Xing, Y., Lubischer, J.L., Krieg, P.A., Krupenko, S.A., *et al.* (2008). A Transcriptome Database for Astrocytes, Neurons, and Oligodendrocytes: A New Resource for Understanding Brain Development and Function. J Neurosci *28*, 264-278.

Castranio, T., and Mishina, Y. (2009). Bmp2 is required for cephalic neural tube closure in the mouse. Developmental Dynamics *238*, 110-122.

Conti, L., Pollard, S.M., Gorba, T., Reitano, E., Toselli, M., Biella, G., Sun, Y., Sanzone, S., Ying, Q.L., Cattaneo, E., *et al.* (2005). Niche-independent symmetrical self-renewal of a mammalian tissue stem cell. PLoS biology *3*, e283.

Hochberg, Y., and Benjamini, Y. (1990). More powerful procedures for multiple significance testing. Statistics in medicine *9*, 811-818.

Huang da, W., Sherman, B.T., and Lempicki, R.A. (2009). Systematic and integrative analysis of large gene lists using DAVID bioinformatics resources. Nature protocols *4*, 44-57.

Irizarry, R.A., Hobbs, B., Collin, F., Beazer-Barclay, Y.D., Antonellis, K.J., Scherf, U., and Speed, T.P. (2003). Exploration, normalization, and summaries of high density oligonucleotide array probe level data. Biostatistics *4*, 249-264.

Jackson, S.P., and Bartek, J. (2009). The DNA-damage response in human biology and disease. Nature *461*, 1071-1078.

Obayashi, S., Tabunoki, H., Kim, S., and Satoh, J.-i. (2009). Gene Expression Profiling of Human Neural Progenitor Cells Following the Serum-Induced Astrocyte Differentiation. Cellular and Molecular Neurobiology *29*, 423-438.

Sabapathy, K., Klemm, M., Jaenisch, R., and Wagner, E.F. (1997). Regulation of ES cell differentiation by functional and conformational modulation of p53. The EMBO journal *16*, 6217-6229.

Smyth, G.K. (2004). Linear models and empirical bayes methods for assessing differential expression in microarray experiments. Statistical applications in genetics and molecular biology *3*, Article3.

Spiliotopoulos, D., Goffredo, D., Conti, L., Di Febo, F., Biella, G., Toselli, M., and Cattaneo, E. (2009). An optimized experimental strategy for efficient conversion of embryonic stem (ES)-derived mouse neural stem (NS) cells into a nearly homogeneous mature neuronal population. Neurobiology of Disease *34*, 320-331.

Taga, T., and Fukuda, S. (2005). Role of IL-6 in the neural stem cell differentiation. Clinical Reviews in Allergy and Immunology *28*, 249-256.

Turnley, A.M., and Bartlett, P.F. (2000). Cytokines that Signal Through the Leukemia Inhibitory Factor Receptor-β Complex in the Nervous System. Journal of neurochemistry *74*, 889-899.

Xu, Y., and Baltimore, D. (1996). Dual roles of ATM in the cellular response to radiation and in cell growth control. Genes & development *10*, 2401-2410.
